# Engineering prokaryotic regulator IrrE to enhance stress tolerance in budding yeast

**DOI:** 10.1186/s13068-020-01833-6

**Published:** 2020-11-30

**Authors:** Li Wang, Xin Wang, Zhi-Qiang He, Si-Jie Zhou, Li Xu, Xiao-Yu Tan, Tao Xu, Bing-Zhi Li, Ying-Jin Yuan

**Affiliations:** 1 Frontiers Science Center for Synthetic Biology and Key Laboratory of Systems Bioengineering (Ministry of Education), School of Chemical Engineering and Technology, Tianjin University, Tianjin, 300072 P.R. China; 2 Synthetic Biology Research Platform, Collaborative Innovation Center of Chemical Science and Engineering (Tianjin), Tianjin University, Tianjin, 300072 P.R. China; 3 State Key Laboratory of Materials-Oriented Chemical Engineering, College of Biotechnology and Pharmaceutical Engineering, Nanjing Tech University, Nanjing, 211816 Jiangsu P.R. China

**Keywords:** IrrE, Global regulatory protein engineering, Lignocellulose-derived inhibitors, Genome-wide transcriptional perturbation, Thermal tolerance

## Abstract

**Background:**

Stress tolerance is one of the important desired microbial traits for industrial bioprocesses, and global regulatory protein engineering is an efficient approach to improve strain tolerance. In our study, IrrE, a global regulatory protein from the prokaryotic organism *Deinococcus radiodurans*, was engineered to confer yeast improved tolerance to the inhibitors in lignocellulose hydrolysates or high temperatures.

**Results:**

Three IrrE mutations were developed through directed evolution, and the expression of these mutants could improve the yeast fermentation rate by threefold or more in the presence of multiple inhibitors. Subsequently, the tolerance to multiple inhibitors of single-site mutants based on the mutations from the variants were then evaluated, and 11 mutants, including L65P, I103T, E119V, L160F, P162S, M169V, V204A, R244G, Base 824 Deletion, V299A, and A300V were identified to be critical for the improved representative inhibitors, i.e., furfural, acetic acid and phenol (FAP) tolerance. Further studies indicated that IrrE caused genome-wide transcriptional perturbation in yeast, and the mutant I24 led to the rapid growth of *Saccharomyces cerevisiae* by primarily regulating the transcription level of transcription activators/factors, protecting the intracellular environment and enhancing the antioxidant capacity under inhibitor environments, which reflected IrrE plasticity. Meanwhile, we observed that the expression of the wild-type or mutant IrrE could also protect *Saccharomyces cerevisiae* from the damage caused by thermal stress. The recombinant yeast strains were able to grow with glucose at 42 ℃.

**Conclusions:**

IrrE from *Deinococcus radiodurans* can be engineered as a tolerance-enhancer for *Saccharomyces cerevisiae*. Systematic research on the regulatory model and mechanism of a prokaryotic global regulatory factor IrrE to increase yeast tolerance provided valuable insights for the improvements in microbial tolerance to complex industrial stress conditions.

## Background

Concerns about energy supplies and global climate change have led to growing attention worldwide for producing biochemicals, biofuels, and biomaterials from lignocellulosic biomass [1, 2]. To officially ferment lignocellulosic hydrolysates, it is highly desired to improve strain tolerance to specific stresses, such as the inhibitors inevitably released from lignocellulose biomass pretreatment and the high temperatures employed in the bioprocess of simultaneous saccharification and ethanol fermentation (SSF) [3, 4]. Studies on genetic analysis on stress tolerance have yielded several beneficial genetic traits associated with furfural or acetic acid tolerance [5–7]. The deletion or overexpression of some resistant structural genes has proven useful for improving strain tolerance to a single inhibitor [8–10]. However, due to the diversity and complexity of inhibitors in lignocellulosic hydrolysates, the tolerance to combined inhibitors becomes more difficult and involves multigenic traits [11]. Phenolic compounds, furan aldehydes, and carboxylic acids are three main groups of inhibitory molecules present in the lignocellulosic pretreated hydrolysates [12]. Furfural, acetic acid, and phenol are the three representations of those three groups. Our group has conducted studies on the tolerance mechanism against multiple inhibitors of FAP and has found increasing proline and myoinositol as the new determinants for improving strain tolerance to FAP [13, 14]. Thus, simultaneously regulating multiple genes may be a feasible route to enhance strain tolerance to complex inhibitors.

Global transcription machinery engineering (gTME) is a directed evolutionary method to improve cellular phenotypes, and a strain library with different phenotypes was generated by using this method [15]. This approach has been successfully explored in reprogramming cellular tolerance to substrates or product phenotypes [15–18]. For example, Alper and his co-workers generated a mutant library of the TATA-binding protein Spt15 and conferred yeast increased ethanol tolerance [17]. In recent years, several global regulators in *Escherichia coli* have been well characterized, such as CRP, FNR, IHF, FIS, ArcA, NarL, and Lrp, which directly regulate the expressions of 51% of genes in *E. coli *[19, 20]. Through the genetic analysis of a DNA damage-sensitive strain *Deinococcus radiodurans*, the regulatory protein IrrE was discovered [21]. The next series of research found that IrrE played an important role in resisting *D. radiodurans*. After radiation, IrrE specifically bound the promoters DNA of pprA and recA, which were encoding genes from DNA repair proteins to stimulate gene transcriptions and expressions [21–24]. More extensively, IrrE up-regulated more than 210 gene transcription levels after radiation, including 21 genes for DNA replication and repair, which strongly indicated that IrrE conferred the *D. radiodurans* radiation resistance ability by regulating many DNA repair and protection pathways [24]. Lately, wild-type or engineered *IrrE* were reported to improve stress tolerances of heterologous hosts. The overexpression or modification of IrrE has been proved to be capable of enhancing *E. coli* tolerance to various stressful conditions, such as ethanol, butanol, salt, furfural, acetic acid, 5-hydroxymethyl-2-furaldehyde (HMF), and osmotic stress [25–29]. The expression of the protein IrrE also protected *Zymomonas mobilis* against ethanol and acid stress [30]. The introduction of IrrE efficiently improved *Arthrobacter simplex* tolerance to organic solvents and various abiotic stresses as well [31]. Recently research exhibited that *Sphingomonas sp.* with the gene *IrrE* and *Pseudomonas putida* possessing the same gene showed enhanced tolerance to acid [32, 33]. IrrE, being reported a global regulator in *E. coli* and *A. simplex*, brought about efficient stress tolerances, to name a few, salt and ethanol stresses [25, 34–36]. In addition to its application in prokaryotes, the expression of IrrE also provided enhanced tolerance to increased salt for *B. napus* and improved resistance toward salt, 5-hydroxymethylfurfural, furfural, formic and acetic acid for *Saccharomyces cerevisiae *[25, 37, 38].

**Fig. 1:**
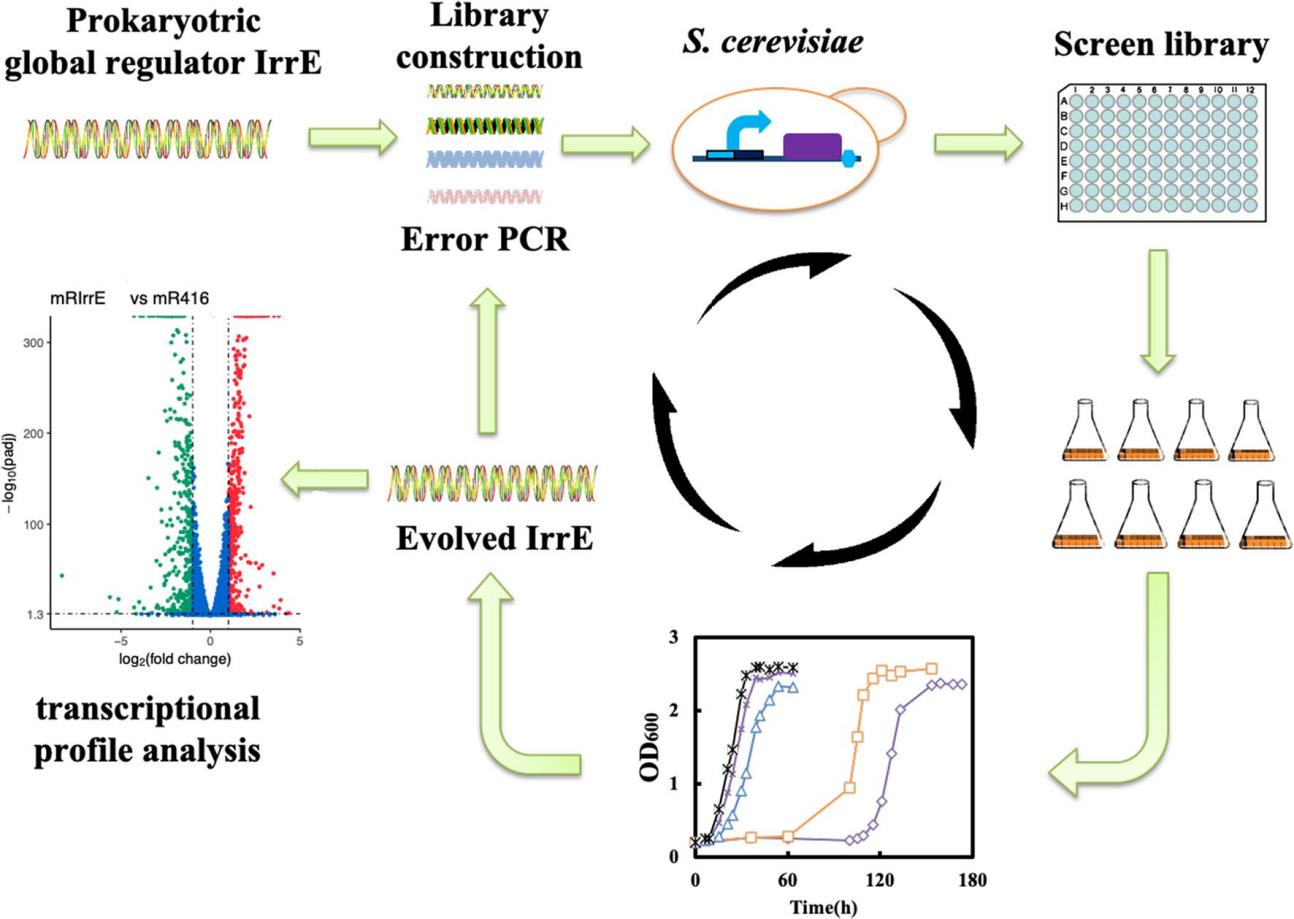
A general schematic for the directed evolution of IrrE to improve microbial tolerance. The strategy of error-prone PCR was carried out to introduce diversity to the genes. The mutant genes are then ligated into the pRS416 vector with HXT7 promoter and TEF1 terminator and transferred into *S. cerevisiae* BY4742. The library is screened with a high-throughput method based on the growth biomass in the presence of FAP. High biomass is related with faster growth and selected for further analysis in flask fermentation. Improved mutants are isolated for the next round of modification and screening. According to the transcriptome analysis, regulatory networks were constructed to reveal the regulatory mechanisms by which IrrE and I24 enhance yeast tolerance

From the aspect of the strain tolerance to FAP, we studied the biological effects and regulatory mechanisms of IrrE on *S. cerevisiae* (Fig. 1). The wild-type IrrE and the mutants obtained by directed evolution were used to improve yeast resistance to FAP and thermal stress. This work emphasizes that the heterologous expression of IrrE played a complex regulatory role in *S. cerevisiae*, which provides new approaches for enhancing cell tolerance.

## Results and discussion

### Expressing IrrE to enhance yeast tolerance to acetic acid

**Fig. 2:**
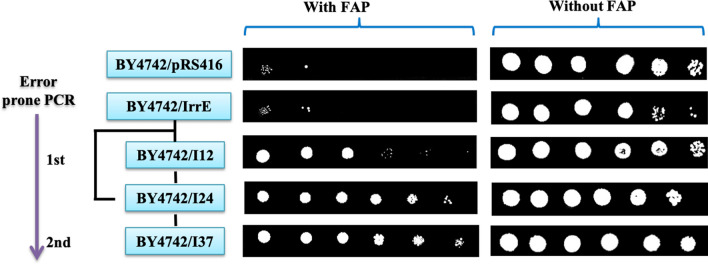
Effect of mutant IrrE on the FAP tolerance of yeast cells. The growth phenotypes of strain BY4742/pRS416, BY4742/IrrE, BY4742/I12, BY4742/I24 and BY4742/I37 were evaluated on SC-Ura plates in the absence and presence of 0.8 g/L furfural, 3.0 g/L acetic acid, and 0.3 g/L phenol

**Fig. 3:**
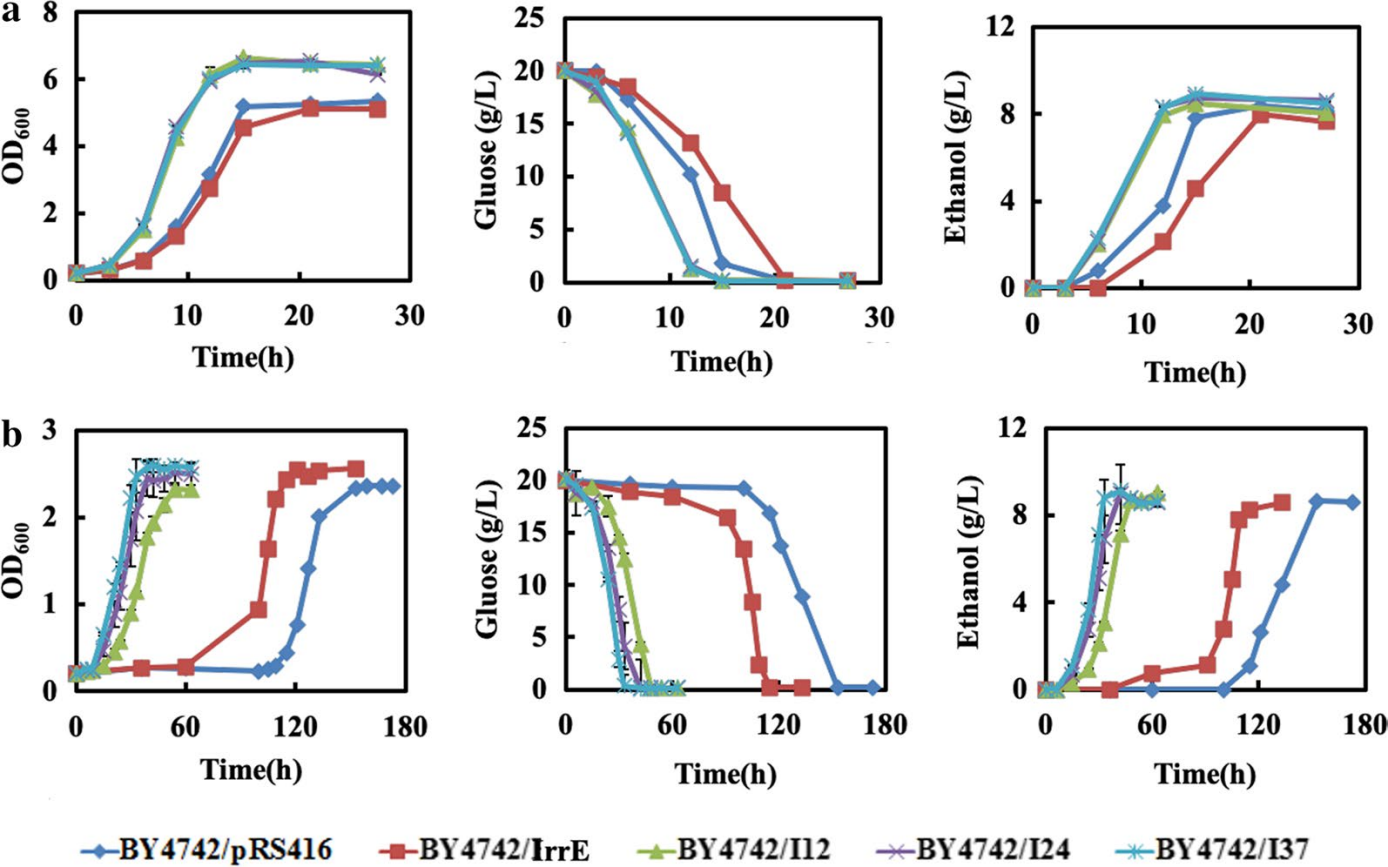
The effect of FAP on fermentation profiles of the strain BY4742/pRS416, BY4742/IrrE, BY4742/I12, BY4742/I24 and BY4742/I37. **a** The growth behaviors, glucose consumption and ethanol production of the strains in the absence of multiple inhibitors. **b** The growth behaviors, glucose consumption and ethanol production of the strains in the presence of 0.8 g/L furfural, 3.0 g/L acetic acid, and 0.3 g/L phenol. Results are the mean of duplicate experiments and error bars indicate s.d

To study the effect of IrrE on the inhibitor tolerance of *S. cerevisiae,* we first evaluated the IrrE role on strain tolerance to acetic acid, a primary inhibitor in lignocellulose hydrolysates. In the presence of acetic acid, the strain BY4742/IrrE entered the exponential phase at about 30 h and reached the stationary phase at about 54 h with the maximal OD_600_ = 3.56. In comparison, the control strain growth was recovered until 48 h and reached a maximum OD_600_ = 3.12 at 67 h (Additional file 1: Fig. S1). The results showed that the heterologous expression of IrrE slightly enhanced the ability of *S. cerevisiae* cells to resist acetic acid shock, but it did not confer to strains the desired acid tolerance. To further verify the tolerance of strain BY4742/IrrE relative to strain BY4742/pRS416 to FAP, cell viability was tested. The strain BY4742/IrrE did not display a clear increase in cell viability when compared with the control strain in the presence of inhibitors. However, the spot assay shows a slight decrease in cell viability of BY4742/IrrE in the absence of inhibitors (Fig. 2). Further, we determined the growths of strain BY4742/IrrE and strain BY4742/pRS416 in the presence of FAP, and strain BY4742/IrrE grew much better in the presence of FAP (Fig. 3b). Previous studies have shown that the expression of engineered IrrE can enhance *Zymomonas mobilis* and yeast acetic acid tolerance [30, 37]. Therefore, it is necessary to optimize IrrE in *S. cerevisiae* to improve FAP tolerance further.

### Directed evolution of IrrE to improve strain tolerance

The directed evolution strategy has been widely used in protein modifications and showed that engineered IrrE conferred *E. coli* enhanced tolerances toward lignocellulosic hydrolysates inhibitors [27]. To further optimize strain tolerance to lignocellulose-derived inhibitors, especially the mixture of multiple inhibitor FAP, directed evolution was employed to modify IrrE in *S. cerevisiae*. As described in the part of methods, the *IrrE* library of about 10^5^ mutants was generated. The transformants were initially selected in the 96-well plates based on strain growth in the SC-Ura medium with FAP (Fig. 1). The growth rates of the isolated mutants were further verified in the tube and flask (Fig. 1).

Accordingly, in the first round of mutagenesis and selection, two mutants BY4742/I12 and BY4742/I24, were isolated. Compared to the strain BY4742/pRS416 and the strain BY4742/IrrE, significant growth increases of the two mutants were observed in the presence of FAP (Fig. 2). Meanwhile, strain BY4742/I24 exhibited higher FAP tolerances than the strain BY4742/I12 (Fig. 2). To further improve performance, the mutant *I24* from strain BY4742/I24 was used as the template for the second round of directed evolution, and the strain BY4742/I37 was obtained. Compared to the strain BY4742/I24, the strain BY4742/I37 growth ability under FAP stress was also slightly improved (Fig. 2). The experiment reflected the potential role IrrE can play in regulating the transcriptional level of genes in *S. cerevisiae*.

### Evolved IrrE to improve strain tolerance to the mixture with multiple inhibitors

As the top level of the hierarchical regulatory network in microorganisms, the global regulators control strain phenotypes by regulating large numbers of gene expressions. The engineering of global regulators has been shown to be a highly efficient approach conferring cells desired for complex phenotypes in both prokaryotic and eukaryotic microbes [14, 17]. This study has evolved IrrE to improve strain tolerance to the mixture with multiple inhibitors. The fermentation abilities of the IrrE mutants were then comparatively analyzed in the SC-Ura medium supplemented with or without multiple inhibitors (0.8 g/L furfural, 3.0 g/L acetic acid and 0.3 g/L phenol). The concentration ratios for multiple inhibitors were according to the composition of lignocellulosic hydrolysates [39]. The conditions of 0.8 g/L furfural, 3.0 g/L acetic acid, and 0.3 g/L phenol were used to distinguish the tolerances of different strains. In the absence of multiple inhibitors, the heterologous expression of *IrrE* in *S. cerevisiae* slightly weakened strain fermentation. The strain BY4742/pRS416 finished fermentation at about 15 h, while 8.5 g/L glucose was still left in the culture of strain BY4742/IrrE (Fig. 3a). A slight decrease in the biomass concentration, specific growth rate, glucose consumption rate, the final ethanol titer, ethanol productivity and ethanol yield were also observed in the strain BY4742/IrrE (Table 1). This result is different from previous studies in bacteria, where IrrE did not cause differences in the growth of strains under normal conditions [25, 31]. However, the mutant of IrrE significantly increased strain biomass yield, glucose consumption rate, specific growth rate, final ethanol titer, ethanol productivity and ethanol yield (Fig. 3a, Table 1).

**Table 1:** Fermentation parameters for the strains carrying the improved IrrE in the presence and absence of FAP. Results represent the mean of duplicate experiments

FAP	Strains	μ (h^−1^)	r_glu_ (g/L/h)	Ethanol titer (g/L)	r_eth_ (g/L/h)	Ethanol yield (%)
−	BY4742/pRS416	0.51 ± 0.01	1.11	8.27 ± 0.18	0.39 ± 0.009	82.6
	BY4742/IrrE	0.45 ± 0.03	0.95	7.97 ± 0.33	0.38 ± 0.015	79.6
	BY4742/I12	0.64 ± 0.02	1.34	8.48 ± 0.16	0.71 ± 0.013	84.7
	BY4742/I24	0.69 ± 0.01	1.34	8.62 ± 0.11	0.72 ± 0.009	86.1
	BY4742/I37	0.67 ± 0.02	1.34	8.57 ± 0.18	0.71 ± 0.015	85.6
+	BY4742/pRS416	0.079 ± 0.02	0.13	8.65 ± 0.13	0.057 ± 0.001	86.4
	BY4742/IrrE	0.075 ± 0.01	0.17	8.27 ± 0.11	0.054 ± 0.001	82.6
	BY4742/I12	0.073 ± 0.01	0.42	8.70 ± 0.14	0.18 ± 0.003	86.1
	BY4742/I24	0.089 ± 0.02	0.48	8.79 ± 0.13	0.18 ± 0.003	87.0
	BY4742/I37	0.10 ± 0.01	0.48	8.80 ± 0.11	0.18 ± 0.002	87.1

When the mixture with three inhibitors was added in the SC-Ura medium, the fermentation performances of five strains were all significantly affected. The first-round mutant strain BY4742/I24 and the second-round mutant strain BY4742/I37 all grew into the stationary phase and exhausted the glucose at about 42 h (Fig. 3b). The first-round mutant BY4742/I12 finished the fermentation within 48 h (Fig. 3b). In contrast, the fermentation of strain BY4742/IrrE was extended to approximately 115 h, while strain BY4742/pRS416 was still in the early exponential phase at that time and finished fermentation until 153 h. The glucose consumption rate and ethanol productivity were in parallel with the growth rate (Fig. 3b). The mutant BY4742/I37 and the mutant BY4742/I24 exhibited the same glucose consumption rate of 0.48 g/L/h, which was about 3.7-fold and 2.8-fold of the strains BY4742/pRS416 and BY4742/IrrE, respectively (Table 1). It should be noted that the remaining glucose in the sample with mutant BY4742/I37 after 33 h was 0.33 g/L while there was 4.13 g/L remaining glucose in that with mutant BY4742/I24. Ethanol productivities for the three mutants were all 0.18 g/L/h, which were about 3.2-fold and 3.3-fold of the strains BY4742/pRS416 and BY4742/IrrE, respectively (Table 1). The aforementioned data demonstrated that although ethanol productivity of the mutants markedly increased, the five strains generated similar ethanol yields in the presence of multiple inhibitors.

### Sequence-based analysis of the mutants

**Fig. 4:**
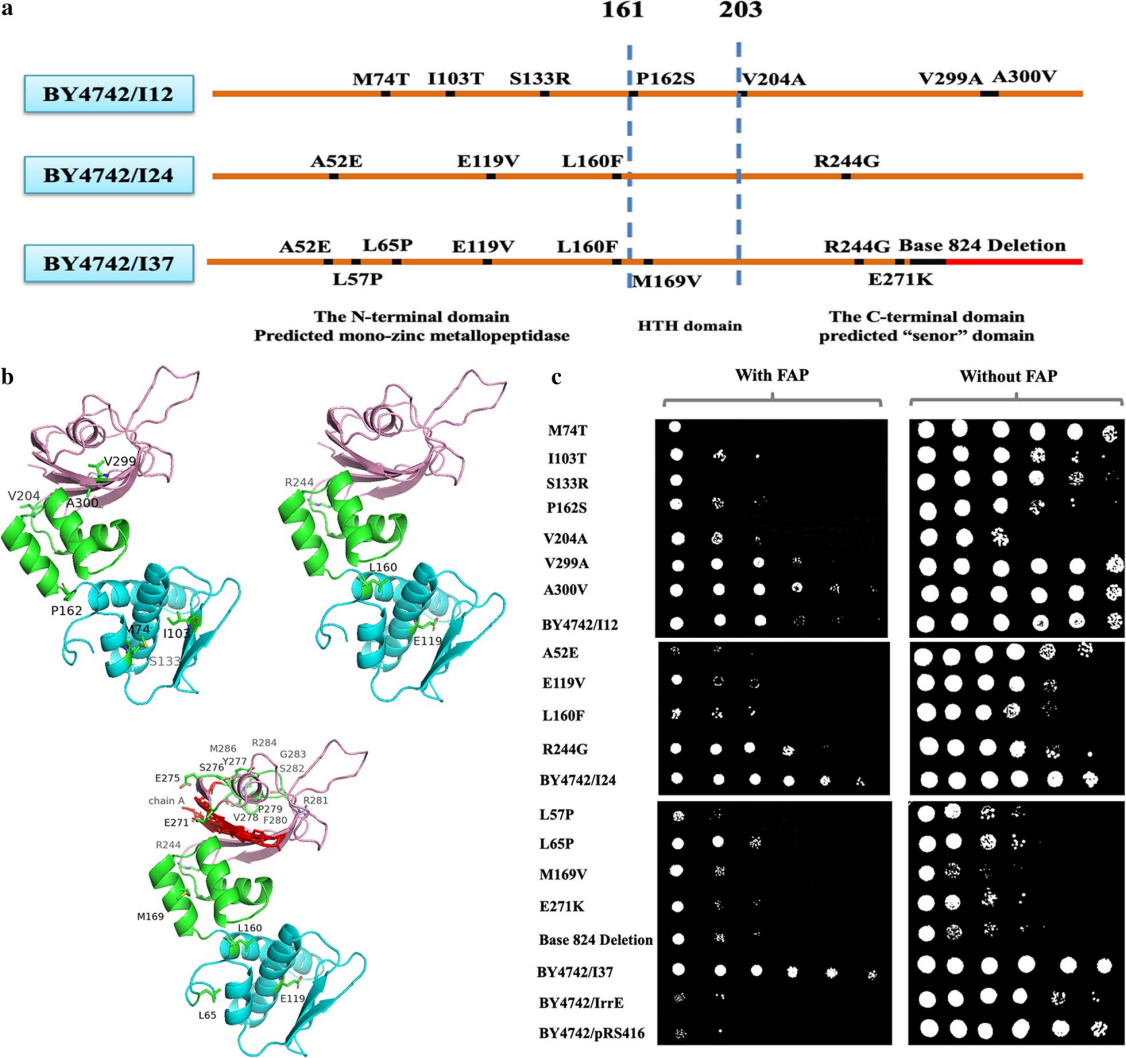
The effect of the single mutation sites on FAP tolerance. **a** The mutation sites of the mutants BY4742/I12, BY4742/I24 and BY4742/I37. **b** Exhibition of mutants BY4742/I12, BY4742/I24 and BY4742/I37 mutation sites in an IrrE modeled structure from *D. radiodurans*. IrrE domains: N-terminal domain colored in cyan; HTH domain colored in green; C-terminal domain colored in pink. **c** The growth phenotypes on SC-Ura plates of *S. cerevisiae* strain BY4742 expressing IrrE variants. The strains were cultivated on SC-Ura plates supplemented without or with 0.8 g/L furfural, 3.0 g/L acetic acid, and 0.3 g/L phenol. The experiments were repeated twice

Based on the aforementioned results, the great tolerance enhancement occurred after two rounds of error-prone PCR. Thus, the three IrrE mutants, I12 and I24 screened from the first round of error-prone PCR, and I37 screened from two rounds of error-prone PCR, were sequenced, and the resultant amino acid sequences were aligned with the wild-type IrrE (Fig. 4a and b). Mutant I12 differed from IrrE at seven amino acid loci, while mutant I24 and mutant I37 had four and nine mutations, respectively. According to the mutations identified in I12, I24, and I37, single-site mutants were generated to investigate their effects on yeast tolerance to multiple FAP (Fig. 4c). For the mutants from I12, I103T, S133R, P162S, and V204A showed a negative impact on strain growth under FAP-free conditions. When the multiple inhibitors were added, the growth of strain I103T, P162S, and V204A exhibited moderate improvement compared to that of strain BY4742/IrrE, and the mutations V299A and A300V significantly increased strain growth ability, while M74T and S133R slightly increased cell viability than the strain BY4742/IrrE. Compared to the mutant I12, A300V was more tolerant to FAP stress, and V299A showed the similar tolerance while I103T and P162S appeared more sensitive. For the four mutants from I24, the growth of E119V and L160F was decreased compared to strain BY4742/IrrE in the FAP-free medium. Under the FAP stress conditions, the growth of A52E was rarely affected compared to that of the strain BY4742/IrrE. In contrast, E119V, L160F, and R244G all rescued strain growth with inhibitors. Meanwhile, these three mutants appeared more sensitive to FAP than the mutant I24, suggesting that these three mutants work synergistically. In addition to the same four mutants as I24 has, I37 has five additional mutants, and their growths all decreased compared to strain BY4742/IrrE in an FAP-free medium. M169V, E271K, and Base 824 Deletion mutants showed a little increased tolerance compared to the control strain under FAP stress conditions, while the mutant L65P showed a moderately enhanced inhibitor tolerance. The improved tolerance of strain I37 may be the result of the combined actions of nine site mutations. Through this comprehensive analysis, 11 mutants of the IrrE protein, including L65P, I103T, E119V, L160F, P162S, M169V, V204A, R244G, Base 824 Deletion, V299A, and A300V, were identified with an essential role in yeast FAP tolerance. As the reported structure of *Deinococcus deserti* IrrE, three domains were identified, including one zinc peptidase-like domain in the N-terminal domain, one helix–turn–helix motif in the middle domain, and one GAF-like domain in the C-terminal domain [40]. Based on the sequence alignment and the homology model, the domain boundaries of *D. radiodurans* IrrE were determined to be: the N-terminal domain covering residues 1–161; the middle domain covering residues 162–203; and the C-terminal domain covering residues 204–328 [26]. Through the mutant analysis, Vujicic-Zagar et al. indicated that the first and third domains are also critical regions for radiotolerance in strain *D. deserti *[40]*.* In this research, except for P162S and M169V, other mutations identified in the strains BY4742/I12, BY4742/I24, and BY4742/I37 all presented at the first and third domains, suggesting the critical role of these two domains in regulating *S. cerevisiae* to tolerant FAP stresses.

### Uncovering the global perturbation generated by IrrE in *S. cerevisiae* in response to the mixture with multiple inhibitors

To study IrrE regulation mechanisms in *S. cerevisiae* cells in response to the mixture of multiple inhibitors, transcriptome sequencing and metabolite analysis were carried out. With or without multiple inhibitors (0.8 g/L furfural, 3.0 g/L acetic acid and 0.3 g/L phenol), samples in the middle of the lag phase were collected for RNA-seq to compare transcriptional profile changes. As a guide, the GO (Gene Ontology) and KEGG (Kyoto Encyclopedia of Genes and Genomes) pathway enrichment analysis of the differentially expressed genes under inhibitor condition were carried out to better reveal the regulatory mechanism of IrrE (Additional file 1: Fig. S2). Following the published method [24], we focused on genes that displayed more than twofold (log_2_foldchange > 1.0 and *p* value < 0.05) increases or decreases in treated BY4742/IrrE strains compared with treated BY4742/pRS416 strain, but not in untreated BY4742/IrrE strains compared with untreated BY4742/pRS416 strains to identify genes related to IrrE regulation in response to FAP. Also, we tested changes in some intracellular metabolites to understand how they responded to FAP stresses. IrrE may regulate the external defenses and internal repair systems to increase strain inhibitors tolerances.

**Fig. 5:**
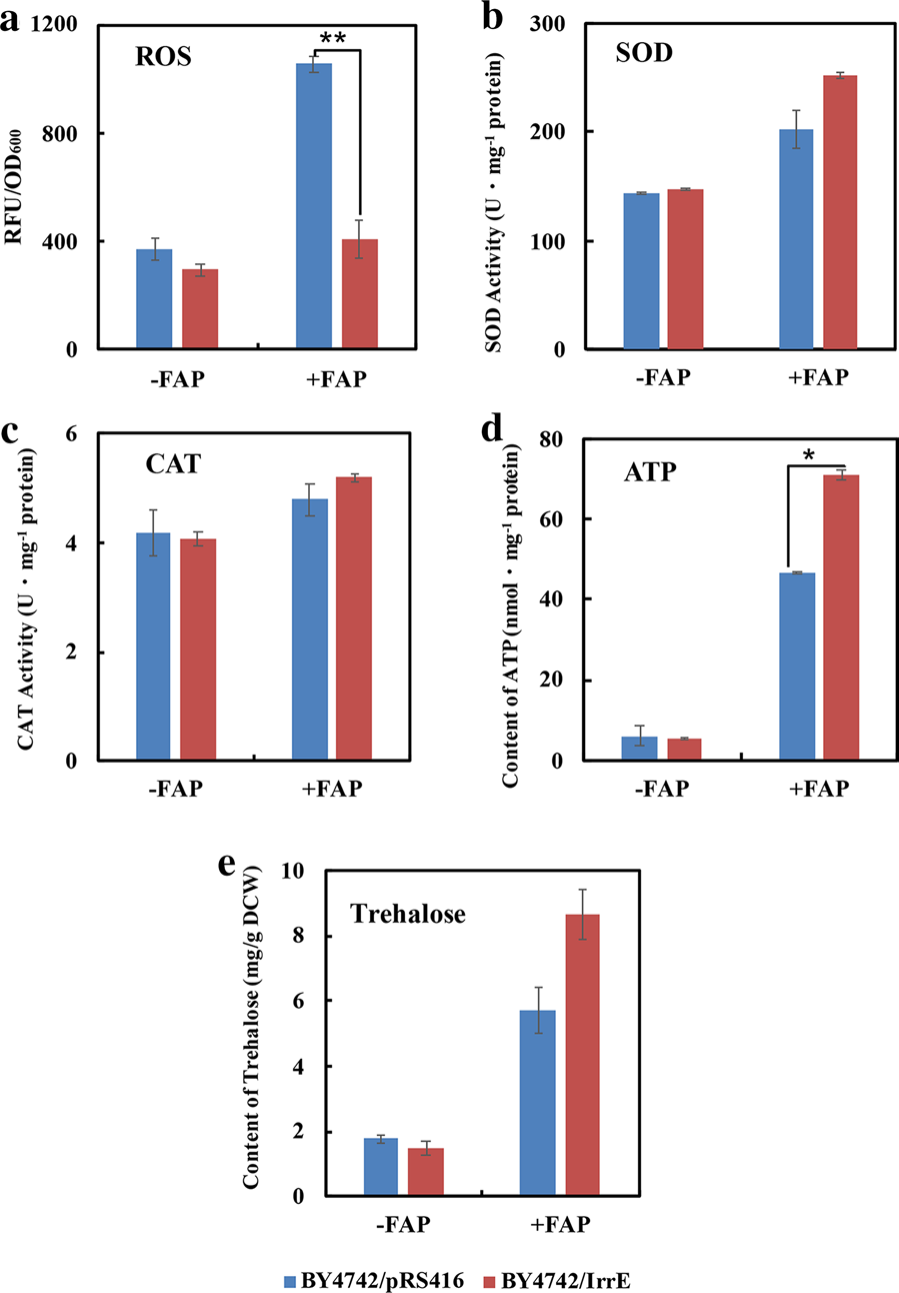
Effect of IrrE on the tolerance related metabolites in *S. cerevisiae*. Intracellular reactive oxygen species (ROS) content (**a**), the activities of SOD (**b**) and CAT (**c**), the intracellular ATP content (**d**) and trehalose content (**e**) in *S. cerevisiae* strains in their middle of the lag phase with or without FAP conditions. Results are the mean of duplicate experiments and error bars indicate s.d. * is for *p* < 0.05 and **is for *p* < 0.01

Acetic acid, furfural, and phenol are the main components of inhibitors produced by lignocellulose pretreatment and have been proven to cause accumulation of reactive oxygen species (ROS) in yeast [10, 41]. As shown in Fig. 5a, the ROS level in untreated strain BY4742/IrrE was slightly lower than that in the untreated strain BY4742/pRS416. However, the ROS level in strain BY4742/IrrE was reduced by about 61.4% compared to that in the control strain after being exposed to FAP, despite the simultaneous increases of ROS levels in both strains. These results implied that the strain BY4742/IrrE suffered less ROS damage. The same protection role of IrrE was also verified in *E. coli* and *Brassica napus* under salt shock and in *E. coli* and *A. simplex* under ethanol shock [21, 25, 35, 42]. The transcription levels of gene encoding enzymes related to ROS detoxification were up-regulated in treated strain BY4742/IrrE (Additional file 2: Table S1). We further determined the activities of superoxide dismutases and catalases, and the results showed that these ROS scavenging enzymes exhibited higher activity in the strain BY4742/IrrE when compared to strain BY4742/pRS416 in the presence of FAP (Fig. 5b and c). These results indicated that the IrrE might reduce intracellular ROS levels by increasing the activities of the related enzymes.

**Fig. 6:**
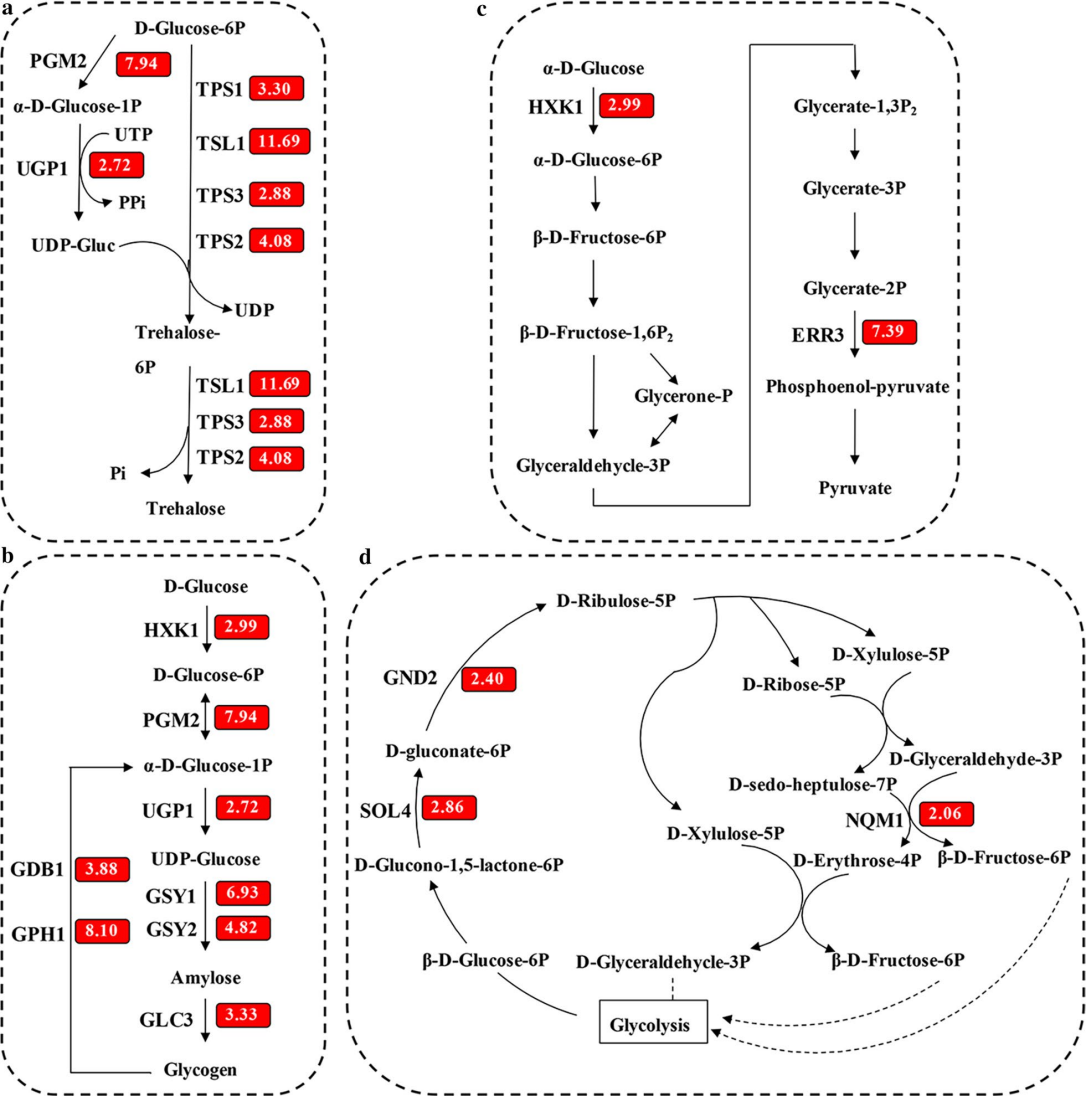
Effect of IrrE on yeast transcription. Transcriptional profiles of accumulation of pressure protectors (**a** and **b**) and energy (**b**), energy metabolism (**c**) and NADPH supply (**d**) by expressing IrrE. The strain BY4742/IrrE and control strain BY4742/pRS416 were cultured in SC-Ura medium with FAP tolerance. Samples were taken in the middle of the lag phase. Box number exhibits transcriptional change, which is the foldchange of the transcriptional level of the strain BY4742/IrrE to that of the control strain BY4742/pRS416. Up-regulated genes were highlighted in red

NADPH is necessary for the process of furfural detoxification in *S. cerevisiae *[43]. The pentose phosphate pathway (PPP) is the primary pathway for the production of NADPH in cells and the transcriptome results showed that the PPP-related genes were up-regulated in treated strain BY4742/IrrE (Fig. 6d), which is accordance with the higher tolerance to FAP of the strain BY4742/IrrE.

The gene *HXK1* and *ERR3* involved in the glycolysis pathway were up-regulated in the treated strain BY4742/IrrE, and the gene *HXK1* encodes the rate-limiting enzyme HXK1 in this pathway (Fig. 6c). Glycolysis is an energy-producing pathway under anaerobic fermentation conditions, and ATP is the main form of energy in yeast cells. The detoxification of inhibitors is an energy-consuming process for yeast, and enhancing energy supplies seems to be necessary for yeast to resist FAP damage. The strain BY4742/IrrE showed a higher ATP yield than the strain BY4742/pRS416 in the presence of FAP (Fig. 5d).

Gene expressions related to trehalose and glycogen biosynthesis were up-regulated in strain BY4742/IrrE under FAP stress (Fig. 6a and b). Trehalose acts as a storage factor and stress protectant in yeast cells. Yeast can synthesize glycogen in response to the stress conditions. In agreement with the transcriptome analysis, the metabolite analysis showed that the trehalose content of the strain BY4742/pRS416 was 20.0% higher than that of the strain BY4742/IrrE under unstressed conditions. In comparison, the trehalose content in the strain BY4742/IrrE was boosted by 51.6% compared to that of the strain BY4742/pRS416 under FAP stress (Fig. 5e). In line with our results, the trehalose contents were significantly increased in the *E. coli* strain with IrrE under osmotic or salt stresses [28, 35]. To summarize, the accumulations of trehalose and glycogen were significantly involved in the IrrE mechanism to improve strain tolerance. Moreover, gene transcriptions related to glycogen degradation was also up-regulated (Fig. 6b), which suggests that the accumulated glycogen can be phosphorylated and then enters the glycolysis pathway to release energy.

Meanwhile, the transcriptome results showed that some sets of genes were also up-regulated in the treated strain BY4742/IrrE, including genes related to DNA repair, some transcription factors/activators, some gene encoding membrane proteins and transport proteins, and some genes associated with the ribosome (Additional file 1: Table S2). These sets of genes should play essential roles in FAP stress resistance for strains with IrrE.

**Fig. 7:**
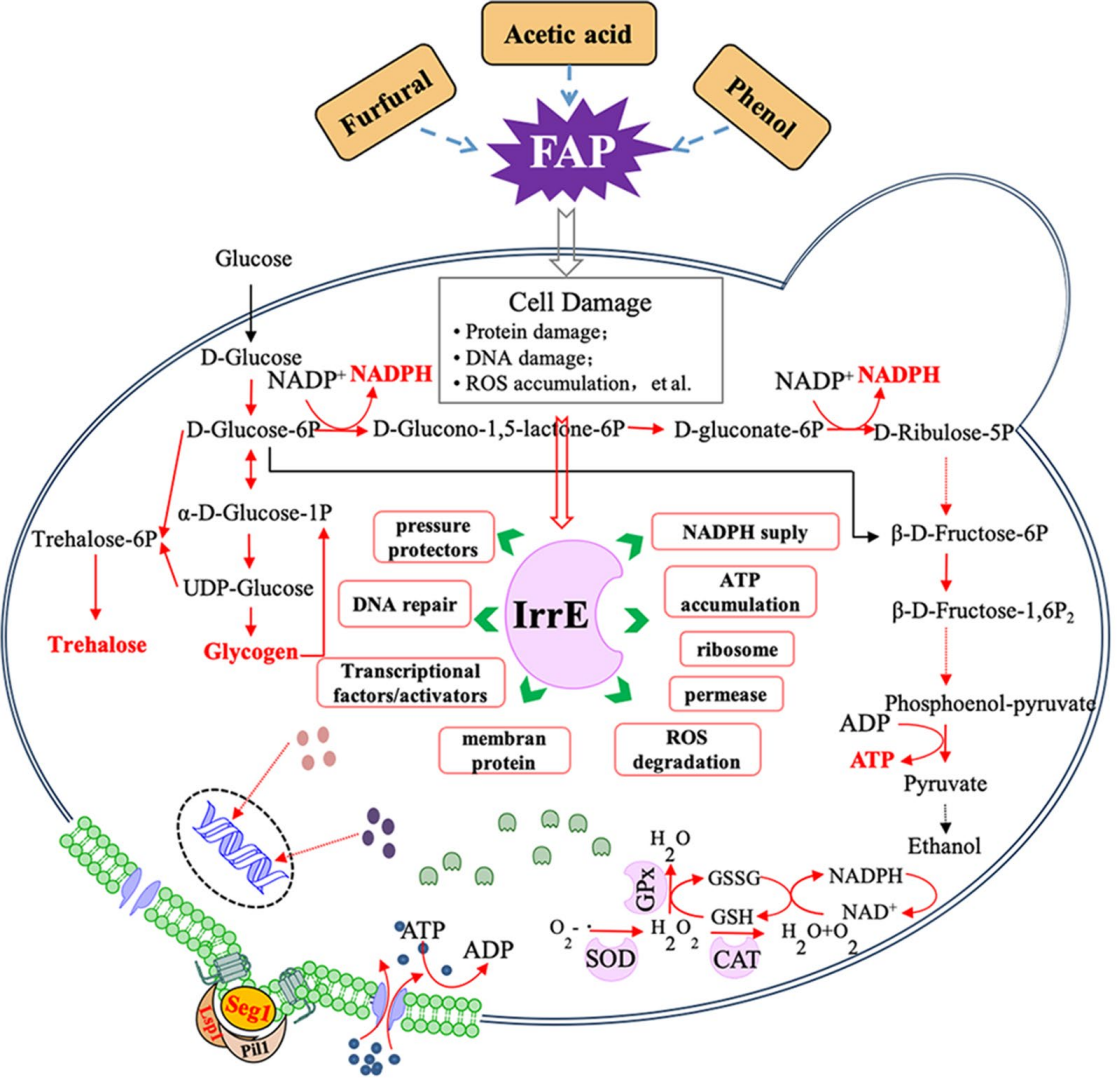
Hypothesis on the mechanisms of IrrE on the enhancement of tolerance to FAP.

From the transcriptome results we can see that IrrE regulated 869 ((log_2_foldchange > 1.0 and *p* value < 0.05) genes in *S. cerevisiae* (Additional file 1: Fig. S3), which were much more than the number of genes changed by mutated transcription factor Spt15p(132) and Taf25-3 in the application of gTME in *S. cerevisiae* under unstressed conditions and oxidation stresses, respectively [17, 44]. As illustrated by Fig. 7, the global transcriptional factors of IrrE may switch on diverse yeast defense systems to resist FAP stress. ROS detoxification plays a vital role in enhancing yeast tolerance by reducing oxidative damage caused by FAP. Accumulated glycogen and trehalose act as pressure protectors to enhance yeast tolerance. At the same time, energy is stored in glycogen and released in the form of ATP through the glycolysis pathway for utilizing energy demand pathways, such as substance transport. NADPH produced by PPP can be used as a cofactor that is essential for inhibitor detoxification. Although we have analyzed the regulatory network for IrrE proteins in yeast in response to inhibitors, the specific genes or proteins that IrrE regulates are still unclear and require further study.

### The transcriptional regulation of I24 in yeast

**Fig. 8:**
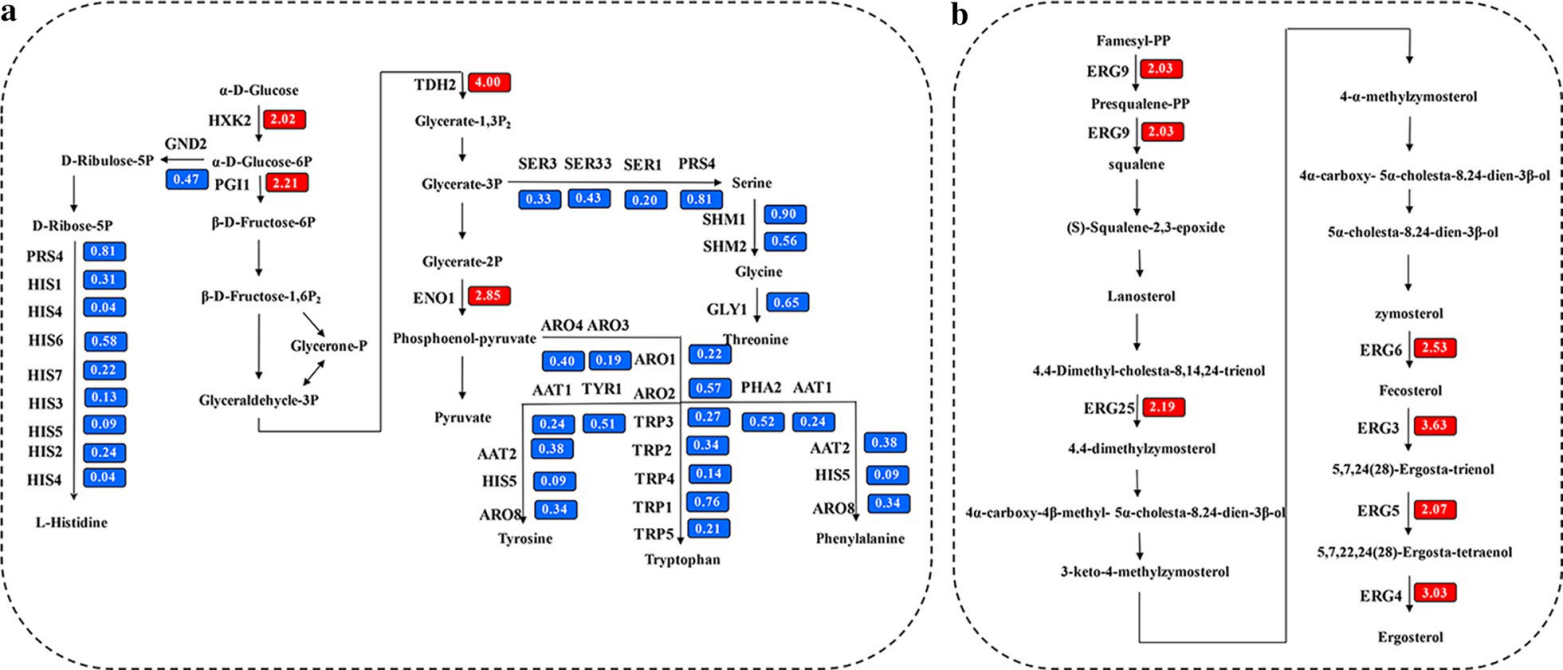
Effect of I24 on yeast transcription. Transcriptional profiles of energy metabolism (**a**) and ergosterol biosynthesis (**b**) by expressing I24. The strain BY4742/I24 and control strain BY4742/pRS416 were cultured in SC-Ura medium without FAP tolerance. Samples were taken in the middle of the lag phase. Box number exhibits transcriptional change, which is the foldchange of the transcriptional level of the strain BY4742/IrrE to that of the control strain BY4742/pRS416. Up-regulated genes were highlighted in red and down-regulated genes were highlighted in blue

Through the directed evolution, three mutant strains with improved tolerance were obtained. The mutant strain BY4742/I24 was selected as a model on which to perform transcriptomic analysis and compared the profile with that of the control strain BY4742/pRS416. We first examined the patterns of the two strains under normal growth conditions. The results of transcriptome analysis proved that the protein I24 might accelerate strain BY4742/I24 growth by modulating genes related to ergosterol biosynthesis and energy metabolism (Fig. 8). Sterols are significant sections of the cytomembrane and are concerned with strain resistance to hydrophobic molecules [45]. From Fig. 8, we can see that most genes associated with ergosterol biosynthesis were up-regulated. Rapid cell growth requires sufficient energy supply. As the regulatory mechanism for IrrE, the transcriptome results of I24 also showed that some genes involved in the glycolysis pathway were up-regulated, and genes related to branch pathways of synthetic amino acids are down-regulated (Fig. 8a).

Like the analytical method for the strain BY4742/IrrE, this mutant group was also performed the GO and KEGG pathway enrichment analysis (Additional file 1: Fig. S4) and then subjected to background removal. The results from transcriptome analysis showed that the protein I24 might enhance the strain BY4742/I24 tolerance by modulating genes in many aspects. Previous studies showed that the entry of acetic acid molecules into cells caused the changes in intracellular pH [46]. Protein PMA1 is necessary for maintaining cytosolic pH homeostasis and the electrochemical potential at the plasma membrane [47–49], and *PMA1* transcription achieved a 2.56-fold time increase in treated strain BY4742/I24, which may be related to the strain BY4742/I24 enhancing tolerance to acetic acid in multiple inhibitors. *D. radiodurans Irr*E protects *Z. mobilis* cells against low levels of pH (pH = 2.0 and pH = 3.5) caused by high levels of acidity [50], which was consistent with our results in yeast. The addition of amino acids enhances the tolerance of *S. cerevisiae* to ethanol and multiple inhibitors. In the meantime, the transcription levels of some amino acid permeases were also shown to be up-regulated (Additional file 1: Table S3), which is in accordance with the higher tolerance to FAP of the strain BY4742/I24. Ribosome biogenesis is the core process for cell growth [51]. Many up-regulated genes were enriched in the ribosome biogenesis set, which demonstrated that the rapid ribosome assembly in treated strain BY4742/I24 is to accommodate protein processing required for rapid growth (Additional file 1: Fig. S5). Furthermore, the protein I24 also enhanced strain tolerance by modulating some translation initiation factors and general stress response elements. Additional file 1: Table S3 shows that three transcription activators and two transcription factors were up-regulated in the treated strain BY4742/I24, among which MSN2 and MSN4 are two associated transcription activators and have become active under many stress conditions [52, 53].

Therefore, the protein I24 may cause a wide range of perturbations by regulating the transcription levels of transcription activators/factors, stabilizing the cell membrane and removing excess H^+^ to protect the intracellular environment, and enhancing the antioxidant capacity under the inhibitor environment. It is suggested that while directed evolution conferred the different regulatory mechanisms to the strain BY4742/I24; on the other hand, it reflected IrrE plasticity.

### IrrE effect on strain thermotolerance

**Fig. 9:**
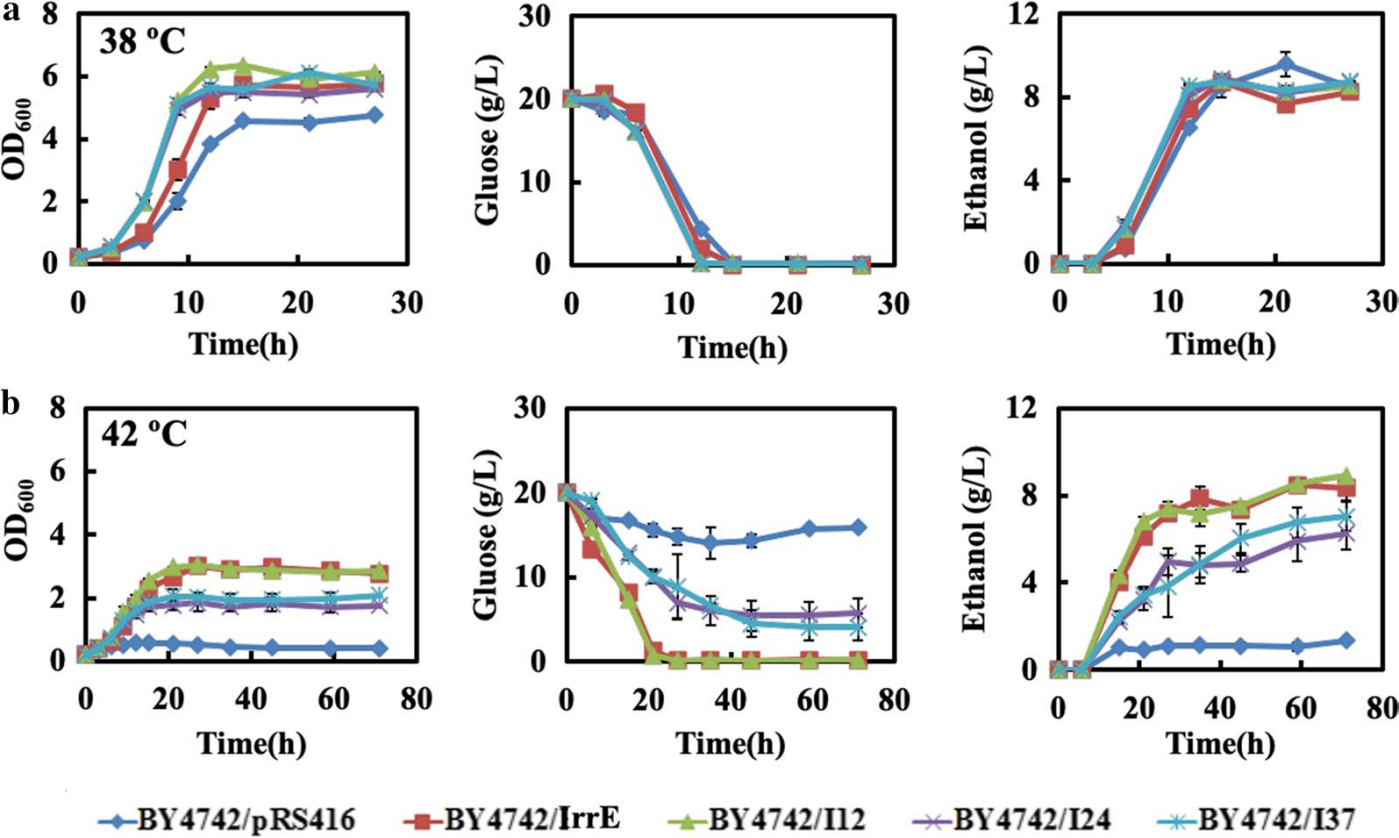
The effect of IrrE mutants on thermal tolerance. The strains were cultivated in SC-Ura medium at (**a**) 38 ºC and (**b**) 42 ºC. Results are the mean of duplicate experiments and error bars indicate s.d.

To explore whether the global regulator IrrE from prokaryotes could elicit the tolerance of yeast cells for high temperatures, which is also necessary in the bioprocessing of simultaneous saccharification and ethanol fermentation (SSF) from lignocellulose. The effects of the wild-type and mutant IrrE on strain thermotolerance were investigated. As shown in Fig. 9, the fermentation abilities of the five strains were compared on growth, glucose consumption and ethanol production in the SC-Ura medium at 38 ºC and 42 ºC. Under the heat stock of 38 ºC, all strains were capable of exhausting glucose in the fermentation medium. The strains expressing wild or mutant IrrE exhibited higher specific growth rates, glucose consumption rates, final ethanol titer, ethanol productivity, and final biomass concentrations than the control strain BY4742/pRS416 (Fig. 9a and Table 2). At 42 ºC, the cell growth, sugar consumption, final ethanol titer, ethanol productivity, and biomass production were all significantly affected regardless of if it was the control strain or the four recombinant strains (Fig. 9b and Table 2). The growth of the control strain BY4742/pRS416 was almost completely suppressed. It had approximately 5 g/L glucose consumed and 1 g/L ethanol produced within 35 h, and no more glucose was subsequently utilized until 72 h. However, an excellent fermentation advantage was observed in recombinant strains. The mutant strain BY4742/I24 and BY4742/I37 could grow into the stationary phase at about 21 h, and approximately 15.5 g/L glucose was consumed in 45 h. The strain BY4742/I37 exhibited a moderate advantage in the maximum biomass, final ethanol titer, ethanol productivity, and final ethanol yield than those in the strain BY4742/I24. The strain BY4742/IrrE and BY4742/I12 exhausted 20 g/L glucose in 21 h and entered the stationary phase with a higher biomass and ethanol production. These results suggested that *S. cerevisi*ae cells could be conferred as to having an enhanced tolerance against thermal stresses through expressing IrrE or its mutants, consistent with the previous findings that IrrE could protect *E. coli* cells from thermal shocks [25].

**Table 2:** Fermentation parameters for the strains harboring the improved IrrE at different cultivation temperature

FAP	Strains	µ (h^−1^)	r_glu_ (g/L/h)	Ethanol titer (g/L)	r_eth_(g/L/h)	Ethanol yield (%)
38 ℃	BY4742/pRS416	0.44 ± 0.03	1.32	8.52 ± 0.18	0.57 ± 0.012	85.0
	BY4742/IrrE	0.56 ± 0.03	1.50	8.73 ± 0.13	0.58 ± 0.008	87.1
	BY4742/I12	0.78 ± 0.01	1.64	8.84 ± 0.06	0.74 ± 0.005	88.3
	BY4742/I24	0.73 ± 0.01	1.65	8.90 ± 0.08	0.74 ± 0.007	88.9
	BY4742/I37	0.76 ± 0.01	1.66	8.76 ± 0.16	0.73 ± 0.013	87.5
42 ℃	BY4742/pRS416	0.03 ± 0.01	0.21	1.33 ± 0.24	0.038 ± 0.007	13.3
	BY4742/IrrE	0.162 ± 0.01	0.89	8.34 ± 0.23	0.14 ± 0.004	83.3
	BY4742/I12	0.163 ± 0.02	0.92	8.90 ± 0.13	0.15 ± 0.002	88.9
	BY4742/I24	0.106 ± 0.03	0.32	6.26 ± 0.76	0.11 ± 0.006	62.5
	BY4742/I37	0.119 ± 0.01	0.34	7.05 ± 0.78	0.12 ± 0.007	70.4

Results represent the mean of duplicate experiments

**Fig. 10:**
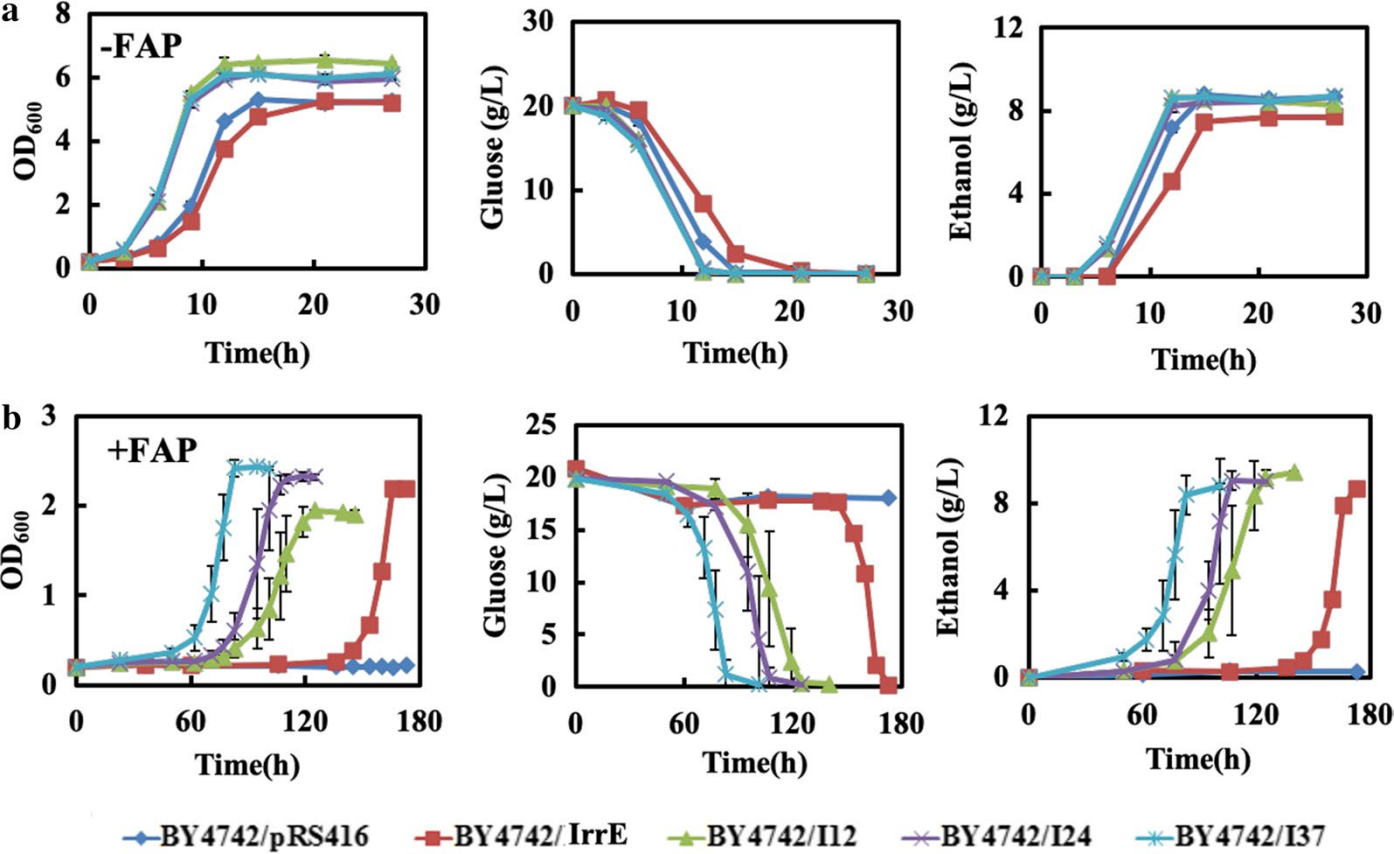
The fermentation profiles of the strain with IrrE mutants under FAP and thermal stress. The growth behaviors, glucose consumption and ethanol production of the strains at 34 ℃ in the absence (**a**) and presence (**b**) of 0.8 g/L furfural, 3.0 g/L acetic acid and 0.3 g/L phenol. Results are the mean of duplicate experiments and error bars indicate s.d

We further evaluated the fermentation performance of the recombinant strains with the wild or mutant IrrE in the presence of multiple inhibitors under high-temperature conditions. When the cultivation temperature was 38 ºC, adding FAP completely suppressed the growth in of all five strains, which were still in the lag phase until 170 h. Rare glucose was consumed and little ethanol was produced (Additional file 1: Fig. S6). Under the condition of 34 ºC, all strains exhibited almost the same growth phenotype with those under the condition of 30 ºC in the absence of FAP (Fig. 10). Similarly, the biomass yield, final ethanol titer, ethanol productivity, glucose consumption rate, and specific growth rate of the three mutants were significantly increased compared to the strain BY4742/pRS416, while the slight decrease of those indicators was observed in strain BY4742/IrrE (Fig. 10). However, at 34 ºC, the presence of FAP significantly enlarged the lag phase and reduced the glucose consumption rate and ethanol productivity. The growth of strain BY4742/pRS416 was still in the lag phase until 170 h, while the strain BY4742/IrrE entered the exponential phase at approximately 135 h (Fig. 10). The strain BY4742/I37, which exhibited the best fermentation capacity, could grow into the stationary phase at about 80 h. The strains BY4742/I12 and BY4742/I24 finished fermentation at about 125 h and 100 h, respectively (Fig. 10). The glucose consumption rate and ethanol productivity were consistent with strain growth (Fig. 10).

We found that the strain BY4742/IrrE exhibited superior performance when compared with the mutant strains with higher tolerances to thermal stresses, suggesting that the mechanism that refers to cellular tolerance to thermal stress differs from that for FAP resistance. We compared the gene transcription level of the strains BY4742/IrrE and strain BY4742/I24 under normal conditions and found that two heat shock protein genes, *HSP12* and *HSP33,* and two key genes related to trehalose synthesis, *TPS3* and *TSL1*, were up-regulated in strain BY4742/IrrE (Additional file 2: Table S1). Heat shock proteins help resist stresses primarily through protecting proteins from misfolding under stress conditions [54, 55]. Additionally, trehalose confers strain tolerance to temperature stress by stabilizing protein activities [56, 57]. The differences in the regulatory mechanisms of the two regulators indicate that IrrE potentially confer yeast a stronger thermal tolerance.

Meanwhile, the different stresses have a synergistically negative effect on cell survival. The increase in fermentation temperature made yeast cells face even more severe challenges under the same concentration of FAP. The synergy of temperature and inhibitors was also observed during simultaneous saccharification and co-fermentation of pretreated corn stover. However, the expression and modification of IrrE could achieve the purpose of enhancing the tolerance of yeast cells to two complex phenotypes (FAP tolerance and thermal tolerance) simultaneously. The modified strains were widely used in simultaneous saccharification and co-fermentation (SScF) process to improve ethanol productions from lignocellulose. Engineered *S. cerevisiae* with xylose metabolizing pathways was used in SScF of aqueous ammonia pretreated corn stover and dry dilute acid-pretreated biomass [58, 59]. Co-fermentation with yeast tolerating inhibitors and yeast utilizing xylose was conducted in the SScF process in undetoxified pretreated biomass to overcome the problem of xylose utilization and inhibitor problems [60]. Co-culture with high-temperature resistant and xylose-utilizing *S. cerevisiae* was developed for ethanol production in temperature-profiled SScF processes [61]. Previous studies indicated that the synergy of high temperature and inhibitors resulted in low viability of the strains in the SScF process [58], and the strain BY4742/IrrE and the mutant strains developed in this study will be helpful for the bioethanol production from SScF of lignocellulose at high temperatures.

## Conclusions

In our study, engineering of global regulatory proteins was conducted to improve the tolerance of yeast cells to the multiple inhibitors that exist in lignocellulose hydrolysates and high temperatures. Through directed evolution of the regulator IrrE from *D. radiodurans* in *S. cerevisiae*, three IrrE mutants with much higher FAP tolerances were developed. The sequence analysis revealed that 11 locus mutations, including L65P, I103T, E119V, L160F, P162S, M169V, V204A, R244G, Base 824 Deletion, V299A, and A300V were critical for enhancing FAP tolerance. We further carried out transcriptome and metabolome analysis and found that IrrE caused global perturbations in *S. cerevisiae* by regulating diverse defense systems to resist FAP stress, including ROS detoxification; NADPH supply; DNA repair; transcription factors/activators; membrane proteins and transport proteins; ribosome proteins; the accumulations of ATP, glycogen, and trehalose. In addition, we also compared the differences between the transcriptomes of I24 in the presence and absence of inhibitors and found that I24 led to the rapid growth of *S. cerevisiae* primarily by regulating the transcription levels of transcription activators/factors, protecting the intracellular environments and enhancing the antioxidant capacity under inhibitor environment. Meanwhile, we observed that the expression of the wild-type or mutant IrrE could also protect *S. cerevisiae* from resisting thermal stresses. Systematic exploration of global regulations in yeast with a prokaryotic global regulatory factor IrrE offers valuable insights for the improvement of microbial tolerance to complex industrial stress conditions.

## Methods

### Strains and plasmids

Strains used in this study are shown in Additional file 1: Table S4. *S. cerevisiae* BY4742 (MATα, his3, leu2, lys2, and ura3) were used for constructing the recombinant strains. *E. coli* DH5α was used for gene cloning and plasmid construction.

To construct the wild-type plasmid pRS416-HXT7p-IrrE-TEF1t, the HXT7 promoter and TEF1 terminator were amplified from *S. cerevisiae S288C,* respectively, using primers HXT7-F/HXT7-R and TEF1t-F/TEF1t-R. The HXT7 promoter was digested with BamHI and EcoRI, and the TEF1 terminator was digested with SalI and XhoI. The gene *IrrE* from *D. radiodurans* was reconstructed with PCR to match the codon preferences of *S. cerevisiae* using primers IrrE-F/IrrE-R*.* After purification and digestion with EcoRI and SalI, the gene *IrrE* was ligated into the pRS416 vector with HXT7 promoter and TEF1t terminator, respectively, to yield plasmid pRS416-HXT7p- IrrE-TEF1t (Additional file 1: Fig. S7). The primers used for plasmid construction are shown in Additional file 1: Table S5.

### Media and culture condition

Yeast strains were cultivated in a liquid SC-Ura medium (synthetic complete medium without uracil; 6.7 g/L yeast nitrogen base without amino acids, 20 g/L glucose, 0.1 g/L leucine, 0.02 g/L histidine, and 0.02 g/L tryptophan). The Luria–Bertani medium (10 g/L peptone, 5 g/L yeast extract, and 5 g/L sodium chloride) containing 100 mg/L ampicillin was used to cultivate *E. coli.*

### Directed evolution of IrrE

The mutant library of the gene *IrrE* was constructed by error-prone PCR, and transformed into the yeast strain BY4742 for screening the mutants with improved inhibitor tolerances. Plasmid pRS416-HXT7p-IrrE-TEF1t was used as the template for the first-generation mutagenesis. Twenty nanograms of DNA template was added to a solution containing 7.0 mM MgCl_2_, 0.2 mM dATP, 0.2 mM dGTP, 0.5 mM dTTP, 0.5 mM dCTP, 1.0 μM primer IrrE-F, 1.0 μM primer IrrE-R, 1 μl of Taq DNA polymerase (5U/μl), 0.35 mM MnCl_2_, and distilled water to make the final volume of 100 μl. The error-prone PCR products were purified, digested with EcoRI and SalI, and inserted into the linearized plasmid pRS416-HXT7p-IrrE-TEF1t digested by the same restriction endonuclease to replace the wild-type *IrrE* gene. The ligation products were transformed into *S. cerevisiae* BY4742 using the lithium acetate/single-stranded carrier DNA/PEG method, and then plated on SC-Ura-agar plates for selection. The library size was approximately 10^5^. Subsequently, approximately 40 groups of screening by 96-well plates with an SC-Ura medium containing 0.8 g/L furfural, 3.0 g/L acetic acid, and 0.3 g/L phenol have been conducted, and 60 strains with shorter lag periods on the growth curve compared with the BY4742/pRS416 strain were obtained to perform tube and flasks fermentations, respectively. According to the length of the lag period of the growth curve and the final OD_600_ of the mutant strains against BY4742/pRS416 strain in the inhibitor environment, the mutant strain BY4742/I12 and strain BY4742/I24 were selected. The process was then repeated for I24 in the second round of mutagenesis. To ensure that the enhanced tolerance was a consequence of the *IrrE* mutated genes, the plasmids containing the mutated *IrrE* were isolated and retransformed.

### Site-directed and insertional mutagenesis

Single-site mutants (A52E, L57P, L65P, M74T, I103T, S133R, E119V, L160F, P162S, M169V, V204A, R244G, E271K, Base 824 Deletion, V299A, A300V) were generated from pRS416-HXT7p-IrrE-TEF1t through two-step PCR procedures to replace wild codons through an overlap extension-PCR protocol. Using the process of constructing the I103T mutant as an example, in step one, two simultaneous PCR reactions were conducted. One fragment was amplified using the primers IrrE-F/I130T-R and the other PCR reactions were performed with a primer pair that included I130T-F and IrrE-R. Then the two fragments were used as the templates and overlapped during the second PCR reaction to obtain full-length mutated IrrE. After gel purification and digestion, the mutated *IrrE* gene was cloned into EcoRI and SalI sites of plasmid pRS416-HXT7p-IrrE-TEF1t replacing the wild-type *IrrE* gene. The ligation products that carried the mutated *IrrE* genes were then transformed into *E. coli* DH5α competent cells to obtain the corresponding plasmids. These plasmids were sequenced to ensure that the correct mutated genes were obtained. The purified plasmids were then transformed into *S. cerevisiae* BY4742 to acquire the corresponding mutants.

### Growth assays

A single colony of yeast strains was first pre-cultured at 30 ºC and 220 rpm for 20 h in the SC-Ura medium. Then, the pre-cultures were transferred into 100 mL of an SC-Ura medium with an initial OD_600_ of 0.1, incubating for 20–24 h at 200 rpm and 30 ºC to get the seed cultures. To test the effects of the IrrE module on strain inhibitor tolerances, the seed cultures for the strain BY4742/IrrE and the control strain BY4742/pRS416 were cultivated in the SC-Ura medium (the initial OD_600_ was approximately 0.2) added with 3.2 g/L acetic acid in 250-mL flasks with a working volume of 100 mL at 150 rpm and 30 ºC. To test the inhibitor tolerances of the mutants, the seed cultures, prepared as described above, were diluted to an optical density at 600 nm of 0.1, and 5 µl aliquots of a tenfold dilution series were spotted onto SC-Ura agar plates supplemented with or without 0.8 g/L furfural, 3.0 g/L acetic acid, and 0.3 g/L phenol. The plates were incubated at 30 °C for 3 days. To test mutant thermotolerance, the plates without inhibitors were incubated at 42 °C for 3 days.

### Fermentation

The seed cultures of the control strain BY4742/pRS416, strain BY4742/IrrE, and the mutants were prepared as described above. The fermentation abilities of the yeast strains in the presence of multiple inhibitors were comparatively analyzed. Fermentation was performed in an SC-Ura medium in 250-mL flasks with a working volume of 100 mL at 30 °C and 150 rpm. The SC-Ura medium was supplemented with or without multiple inhibitors – 0.8 g/L furfural, 3.0 g/L acetic acid, and 0.3 g/L phenol. To test the fermentation abilities of yeast strains at different temperatures, the seed cultures were incubated in 100 mL of an SC-Ura medium at 150 rpm. The fermentation temperature was maintained at 30 °C, 34 °C, 38 °C, and 42 °C. The initial cell densities of fermentation were adjusted to OD_600_ at approximately 0.2. Rubber stoppers were used to cap the flasks to maintain a microaerophilic condition.

### Transcriptome analysis of *S. cerevisiae* strains by RNA-seq

The seed cultures of the control strain BY4742/pRS416, strain BY4742/IrrE, and strain BY4742/I24 were prepared as described above and then were inoculated in an SC-Ura medium supplemented with or without multiple inhibitors – 0.8 g/L furfural, 3.0 g/L acetic acid, and 0.3 g/L phenol, with an initial OD_600_ of 0.2. The cells were grown until the middle of the lag phase at 30 °C in a fermenter (150 rpm) and after that, harvested to perform total RNA isolation using Trizol and was quantified and qualified with the Agilent 2100 Bioanalyzer. Each sample had three biological replicates. The library was generated using NEBNext® Ultra™ RNA Library Prep Kit (Illumina, NEB, USA), and Novogene Inc. conducted sequencing on Illumina HiSeq platform. Clean data (clean reads) were gained from raw reads (raw data) of a FASTQ format through in-house Perl scripts and then used in the downstream analyses. The data were normalized through Htseq software by using RPKM (Reads per Kilo bases per Million reads)-based normalization algorithm [62]. The DESeq software was used to identify differentially expressed genes with log_2_foldchange > 1.0 and *p* value < 0.05. Significantly enriched GO terms and KEGG pathways were selected by comparing with the reference genome with a threshold of *p* value < 0.05. The gene information was found on Saccharomyces genome database (SGD) [63].

### Reactive oxygen species analysis

ROS content was detected with the DCFH-DA staining method [64] with some modifications. Specifically, cells were re-suspended in a phosphate buffer (PBS, pH = 7.0) with a final concentration of 10^7^ cells/mL after washing twice with PBS. Then, adding 10 μg of DCFH-DA (using a 2.5 mg/ml stock dissolved in DMSO) to 1 mL of cell suspension and incubated for 60 min at 30 °C, after which cells were washed twice and re-suspended in 1 mL PBS. The relative fluorescence intensity was measured in a multimode plate reader (SpectraMax M2, Molecular Devices, USA) at an excitation wavelength of 488 nm and an emission wavelength of 525 nm. The OD_600_ value of cell suspension was also measured.

### Antioxidant enzyme activity, trehalose, and ATP determination in *S. cerevisiae* strains

After collection, the cells were washed and re-suspended in the PBS buffer. Then the SOD was analyzed by using the superoxide dismutase (SOD) assay kit (WST-1 method) (Nanjing Jiancheng Bioengineering Institute, Nanjing, China). The catalase (CAT) activity was detected by using the CAT assay kit (Beyotime Biotechnology, Shanghai, China)). The ATP content was measured using an ATP assay kit (Beyotime Biotechnology, Shanghai, China). The protein concentration was measured using the bicinchonininc acid (BCA) protein assay kit (Dingguo Changsheng Biotechnology Co., LTD, Beijing, China) to adjust enzyme activities and ATP content. The intracellular trehalose was extracted and determined by a trehalose content detection kit (Nanjing Jiancheng Bioengineering Institute, Nanjing, China).

### Analytical methods

Spectrophotometer (TU-1810, Beijing, China) was used to measure OD_600_ to monitor cell growth. Glucose and ethanol concentrations were determined by high-performance liquid chromatography (HPLC) using an Aminex HPX-87H ion-exchange column (Bio-Rad, Hercules, CA, USA). The samples were filtered through a 0.2-μM filter before injecting it into the HPLC system. The column was eluted with 5 mM H_2_SO_4_ at a flow rate of 0.6 mL/min at 65 ºC, and detection was performed with the Waters 2414 refractive index detector.

## Supplementary information

**Additional file 1: Table S2.** Up-regulated genes related to FAP stress responses in strain BY4742/IrrE. **Table S3.** Up-regulated genes related to FAP stress responses in the strain BY4742/I24. **Table S4.** Yeast strains and plasmids used in this study. **Table S5.** Primers used in this work with endonuclease restriction sites underlined and italicized as essential. **Fig. S1.** Growth behaviors of strains BY4742/pRS416 and BY4742/IrrE under acetic acid conditions. **Fig. S2.** Representation of differentially expressed genes in selected GO categories and KEGG pathways in the strain BY4742/IrrE after being exposed to multiple inhibitors (0.8 g/L furfural, 3.0 g/L acetic acid and 0.3 g/L phenol) until the middle of the lag phase. (a) The most enriched GO terms of the up-regulated genes. (b) Statistics of pathway enrichment of the up-regulated genes. (c) The most enriched GO terms of the down-regulated genes. (d) Statistics of pathway enrichment of the down-regulated genes. **Fig. S3.** DEGs in the strain BY4742/IrrE in the presence of 0.8 g/L furfural, 3.0 g/L acetic acid and 0.3 g/L phenol. log_2_foldchange > 1.0 and *p* value < 0.05. **Fig. S4.** Representation of differentially expressed genes in selected GO categories and KEGG pathways in the strain BY4742/I24 after being exposed to multiple inhibitors (0.8 g/L furfural, 3.0 g/L acetic acid and 0.3 g/L phenol) until the middle of the lag phase. (a) The most enriched GO terms of the up-regulated genes. (b) Statistics of pathway enrichment of the up-regulated genes. (c) The most enriched GO terms of the down-regulated genes. (d) Statistics of pathway enrichment of the down-regulated genes. **Fig. S5.** Transcriptional profiles of ribosome biogenesis by expressing I24. The strain BY4742/I24 and control strain BY4742/pRS416 were cultured in SC-Ura medium with FAP tolerance. Samples were taken in the middle of the lag phase. Box number exhibits transcriptional changes, which is the foldchange of the transcriptional level of the strain BY4742/I24 to that of the control strain BY4742/pRS416. Up-regulated genes are highlighted in red. **Fig. S6.** The growth behaviors, glucose consumption, and ethanol production of strains BY4742/pRS416, BY4742/IrrE, BY4742/I12, BY4742/I24, and BY4742/I37 at 38 ℃ in the presence of 0.8 g/L furfural, 3.0 g/L acetic acid, and 0.3 g/L phenol. **Fig. S7.** Plasmid map for plasmid pRS416-HXT7p-IrrE-TEF1t and IrrE library construction.

**Additional file 2: Table S1.** Outline of differentially expressed genes in the strains BY4742/IrrE and BY4742/I24 relative to the control strain with or without FAP. log_2_foldchange > 1.0 and *p* value < 0.05.

## Data Availability

The datasets generated during this study are included in this published article and its Additional files 1, 2.

## Abbreviations

*S. cerevisiae*: *Saccharomyces cerevisiae*
*E. coli*: *Escherichia coli*
*D. radiodurans*: *Deinococcus radiodurans*
FAP: Furfural, acetic acid and phenol
SSF: Simultaneous saccharification and ethanol fermentation
HMF: 5-Hydroxymethyl-2-furaldehyde
SC: Synthetic complete medium
SC-Ura: SC medium without uracil
DEGs: Differentially expressed genes
ROS: Reactive oxygen species
SOD: Superoxide dismutase
CAT: Catalases
PPP: Pentose phosphate pathway
GPX: Glutathione peroxidase
DCFH-DA: 2′7′-Dichlorofluorescein diacetate
PBS: Phosphate buffer
