# Supplementary material for: Engineering prokaryotic regulator IrrE to enhance stress tolerance in budding yeast
Source: Biotechnol Biofuels. 2020 Nov 30;13:193. doi: 10.1186/s13068-020-01833-6 (PMC7706047; doi:10.1186/s13068-020-01833-6)
Supplement: Supplementary file 1 — Additional file 1: Table S2. Up-regulated genes related to FAP stress responses in strain BY4742/IrrE. Table S3. Up-regulated genes related to FAP stress responses in the strain BY4742/I24. Table S4. Yeast strains and plasmids used in this study. Table S5. Primers used in this work with endonuclease restriction sites underlined and italicized as essential. Fig. S1. Growth behaviors of strains BY4742/pRS416 and BY4742/IrrE under acetic acid conditions. Fig. S2. Representation of differentially expressed genes in selected GO categories and KEGG pathways in the strain BY4742/IrrE after being exposed to multiple inhibitors (0.8 g/L furfural, 3.0 g/L acetic acid and 0.3 g/L phenol) until the middle of the lag phase. (a) The most enriched GO terms of the up-regulated genes. (b) Statistics of pathway enrichment of the up-regulated genes. (c) The most enriched GO terms of the down-regulated genes. (d) Statistics of pathway enrichment of the down-regulated genes. Fig. S3. DEGs in the strain BY4742/IrrE in the presence of 0.8 g/L furfural, 3.0 g/L acetic acid and 0.3 g/L phenol. log2foldchange > 1.0 and p value < 0.05. Fig. S4. Representation of differentially expressed genes in selected GO categories and KEGG pathways in the strain BY4742/I24 after being exposed to multiple inhibitors (0.8 g/L furfural, 3.0 g/L acetic acid and 0.3 g/L phenol) until the middle of the lag phase. (a) The most enriched GO terms of the up-regulated genes. (b) Statistics of pathway enrichment of the up-regulated genes. (c) The most enriched GO terms of the down-regulated genes. (d) Statistics of pathway enrichment of the down-regulated genes. Fig. S5. Transcriptional profiles of ribosome biogenesis by expressing I24. The strain BY4742/I24 and control strain BY4742/pRS416 were cultured in SC-Ura medium with FAP tolerance. Samples were taken in the middle of the lag phase. Box number exhibits transcriptional changes, which is the foldchange of the transcriptional level of the strain BY4742/I24 to th [file 13068_2020_1833_MOESM1_ESM.docx]

**Additional file 1**

**Table S2.** **Up-regulated genes related to FAP stress responses in strain BY4742/IrrE.**

| Categories | Genes (Fold Change) |
| --- | --- |
| DNA repair | RAD52(3.21)、RAD51(2.22)、RAD14(2.19)、PSO2(2.01)、RAD33(2.08) |
| Transcription factors/ activators | YAP1(2.75)、CIN5(2.90)、RGM1(4.75)、RRN11(2.67)、MSS11(2.28)、TAF11(2.23)、HOT1(2.09)、RPI1(2.77)、MSA2(2.90)、HAA1(2.05) |
| membrane proteins | ATR1(2.88)、LSP1(3.97)、SEG1(2.25)、FMP45(7.20)、YNL194C(5.18)、PST2(2.59)、RTN2(7.41)、TFS1(4.29)、SRC1(3.00)、YME2(2.65)、OM45(2.60)、AIP1(2.49)、HEM15(2.98)、VID24(2.14)、COX14(2.95)、MRL1(2.56)、MCD4(2.19)、YTA12(2.05)、OM14(2.09)、ASI1(2.63)、UIP4(2.33)、TVP18(2.12)、PET111(2.49)、SAM37(2.16)、INP1(2.27)、IMP1(2.03)、LDS1(2.16) |
| transport proteins | AGP1(3.40)、GAP1(3.54)、PNS1(4.51)、TPO3(2.44)、TNA1(2.58)、MUP1(2.74)、MEP2(3.13)、MCH5(2.05)、FET4(2.34)、PTR2(2.23)、UBX2(2.29) |
| Ribosome proteins | MRPL3(2.85)、YML6(2.13)、MRPS17(2.33)、RSM7(2.89)、MRPL33(2.43)、RPL15B(2.19)、MRPS8(3.00)、TMA23(2.08)、MRPL19(2.18)、MRPL51(2.14) |

**Table S3.** **Up-regulated genes related to FAP stress responses in the strain BY4742/I24.**

| Categories | Genes (Fold Change) |
| --- | --- |
| permease | GNP1(3.24)、TPO3(3.12)、GAP1(3.35)、MUP1(3.09)、TPO2(3.97)、AGP1(2.64)、DIP5(2.35)、PUT4(2.23) |
| Translation initiation factor | TIF1(3.93)、TIF4631(2.49)、TIF11(2.42)、TIF3(2.23)、CDC33(2.35)、NIP1(2.54)、RPG1(2.39)、PRT1(2.09)、CLU1(2.46)、RLI1(2.20)、GCN3(2.09) |
| Transcription factors/  activators | MSN4(2.71)、MSN2(2.28)、PHD1(2.24)、MCM1(2.33)、RAP1(2.02) 1(2.09)、CLU1(2.46)、RLI1(2.20)、GCN3(2.09) |
| Membrane related | ELO3(3.42)、PMA1(2.56)、PMP1(3.35) |

**Table S4.** **Yeast strains and plasmids used in this study**

| Yeast strains | Description | Source |
| --- | --- | --- |
| *S. cerevisiae* strains |  |  |
| BY4742 | MATα HIS3 LEU2 LYS2 URA3 | Research Genetics |
| BY4742/pRS416 | BY4742 (pRS416) | This study |
| BY4742/IrrE | BY4742 (pRS416-HXT7p-IrrE-TEF1t) | This study |
| BY4742/I12 | BY4742 (pRS416-HXT7p-IrrE^M74T, I103T, S133R, P162S, V204A, V299A, A300V^-TEF1t) | This study |
| BY4742/I24 | BY4742 (pRS416-HXT7p-IrrE^A52E, E119V, L160F, R244G^ -TEF1t) | This study |
| BY4742/I37 | BY4742 (pRS416-HXT7p-IrrE^A52E, L57P, L65P, E119V, L160F, M169V, R244G, E271K and Base 824 Deletion^-TEF1t) | This study |
| M74T | BY4742 (pRS416-HXT7p-IrrE^M74T^ -TEF1t) | This study |
| I103T | BY4742 (pRS416-HXT7p-IrrE^I103T^-TEF1t) | This study |
| S133R | BY4742 (pRS416-HXT7p-IrrE^S133R^ -TEF1t) | This study |
| P162S | BY4742 (pRS416-HXT7p-IrrE^P162S^ -TEF1t) | This study |
| V204A | BY4742 (pRS416-HXT7p-IrrE^V204A^ -TEF1t) | This study |
| V299A | BY4742 (pRS416-HXT7p-IrrE^V299A^ -TEF1t) | This study |
| A300V | BY4742 (pRS416-HXT7p-IrrE^A300V^-TEF1t) | This study |
| A52E | BY4742 (pRS416-HXT7p-IrrE^A52E^-TEF1t) | This study |
| E119V | BY4742 (pRS416-HXT7p-IrrE^E119V^TEF1t) | This study |
| L160F | BY4742 (pRS416-HXT7p-IrrE^L160F^-TEF1t) | This study |
| R244G | BY4742 (pRS416-HXT7p-IrrE^R244G^-TEF1t) | This study |
| L57P | BY4742 (pRS416-HXT7p-IrrE^L57P^-TEF1t) | This study |
| L65P | BY4742 (pRS416-HXT7p-IrrE^L65P^-TEF1t) | This study |
| M169V | BY4742 (pRS416-HXT7p-IrrE^M169V^-TEF1t) | This study |
| E271K | BY4742 (pRS416-HXT7p-IrrE^E271K^-TEF1t) | This study |
| Base 824 Deletion | BY4742 (pRS416-HXT7p-IrrE^Base 824 Deletion^-TEF1t) | This study |

**Table S5** **Primers used in this work with endonuclease restriction sites underlined and italicized as essential.**

| Primer ID | Sequence (5’-3’) |
| --- | --- |
| HXT7p_F | CCCCCCGGGAGAAGGTTTTGGGACGCTC |
| HXT7p_R | CGGAATTCTTTTTGATTAAAATTAAAAAAACTTTTTG |
| TEF1t_F | ACGCGTCGACAAATAAGGAGATTGATAAGACTTTTC |
| TEF1t_R | CCCTCGAGGGCTAACTCTCAACAGACAACAAC |
| IrrE_F | CGGAATTCATGAAGGATGCTAATGAGAGTAAAT |
| IrrE_R | ACGCGTCGACTCAACGAGGTGGGAATGCC |
| 74_F | ATTCTCTGACGGCCGGTGTT |
| 74_R | AACACCGGCCGTCAGAGAAT |
| 103_F | TATTCTGACCAACTCTGCGGC |
| 103_R | GCCGCAGAGTTGGTCAGAATA |
| 133_F | CCTGCTGAGAGACATCCACG |
| 133_R | CGTGGATGTCTCTCAGCAGG |
| 162_F | CCTCATGTCTGAGCCTGTA |
| 162_R  204_F  204_R | TACAGGCTCAGACATGAGG  AAACCCCGGCTCCTGTTATCTAC  AGATAACAGGAGCCGGGGTTTG |
| 299_F | GTCTCGTGGTATCGCTGCTG |
| 299_R | CAGCAGCGATACCACGAGAC |
| 300_F | TATCGTTGTTGTTAGCTTTG |
| 300_R | CAAAGCTAACAACAACGATA |
| 52_F | AGCGGCGAAGGAGCGTAT |
| 52_R | ATACGCTCCTTCGCCGCT |
| 119_F | CTCGCGCATGTAATCGGTC |
| 119_R | GACCGATTACATGCGCGAG |
| 160_F | GCAGCGATCTTCATGCC |
| 160_R | GGCATGAAGATCGCTGC |
| 244_F | TCTTCTTCTACCGGTGGTG |
| 244_R | CACCACCGGTAGAAGAAGA |
| 57_F | TGCGTGACCCGGCGGCAGCGTAC |
| 57_R | ACGCTGCCGCCGGGTCACGCATAC |
| 65_F | ACGTTGCGGCGCCCCCAGGT |
| 65_R | ACCTGGGGGCGCCGCAACGTAC |
| 169_F | TCGCGGAAGTGCTGGAGCGTTTTG |
| 169_R | AAACGCTCCAGCACTTCCGC |
| 271_F | ACCGGCATGAAAGTTCGTGAAG |
| 271_R | TTCACGAACTTTCATGCCGG |
| 824_F | TGGAAGTTCGTGAAGATCCTATGTTCCTTTC |
| 824_R | AGGAACATAGGATCTTCACGAACTTCC |

**Fig. S1**

**
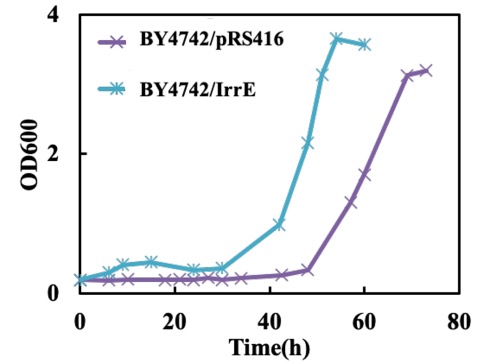
**

Fig. S1: Growth behaviors of strains BY4742/pRS416 and BY4742/IrrE under acetic acid condition.

**Fig. S2**


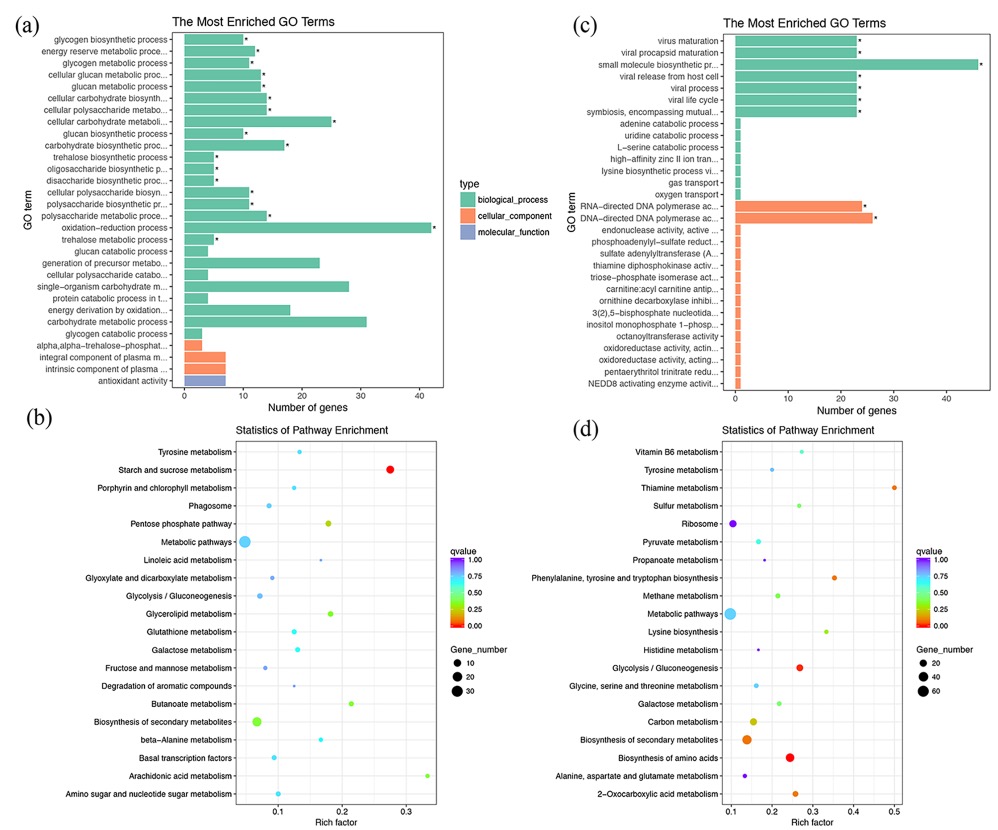


**Fig. S2:** Representation of differentially expressed genes in selected GO categories and KEGG pathways in the strain BY4742/IrrE after being exposed to multiple inhibitors (0.8 g/L furfural, 3.0 g/L acetic acid and 0.3 g/L phenol) until the middle of the lag phase. (a) The most enriched GO terms of the up-regulated genes. (b) Statistics of pathway enrichment of the up-regulated genes. (c) The most enriched GO terms of the down-regulated genes. (d) Statistics of pathway enrichment of the down-regulated genes.

**Fig. S3**


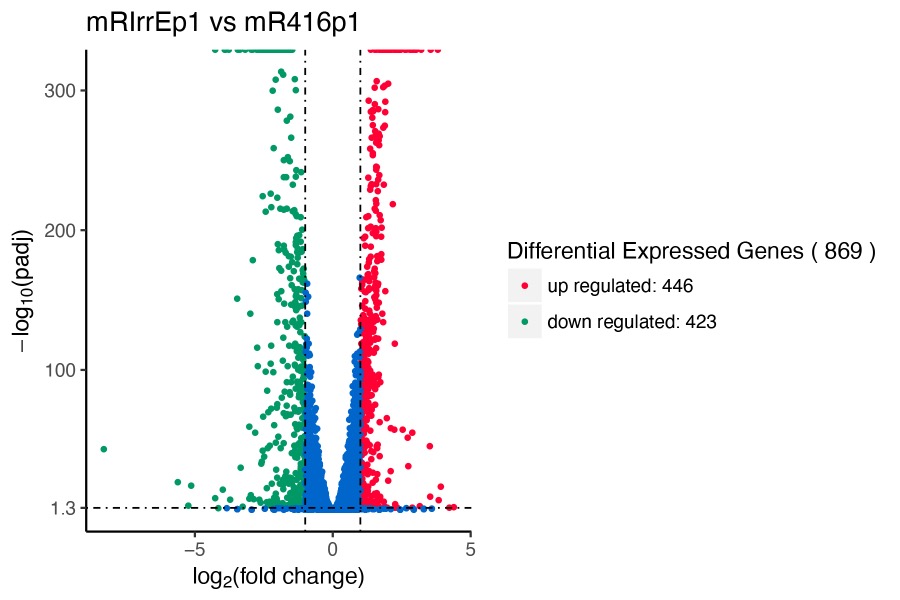


**Fig. S3:** DEGs in the strain BY4742/IrrE in the presence of 0.8 g/L furfural, 3.0 g/L acetic acid and 0.3 g/L phenol. log_2_foldchange>1.0 and *p* value<0.05

**Fig. S4**

**
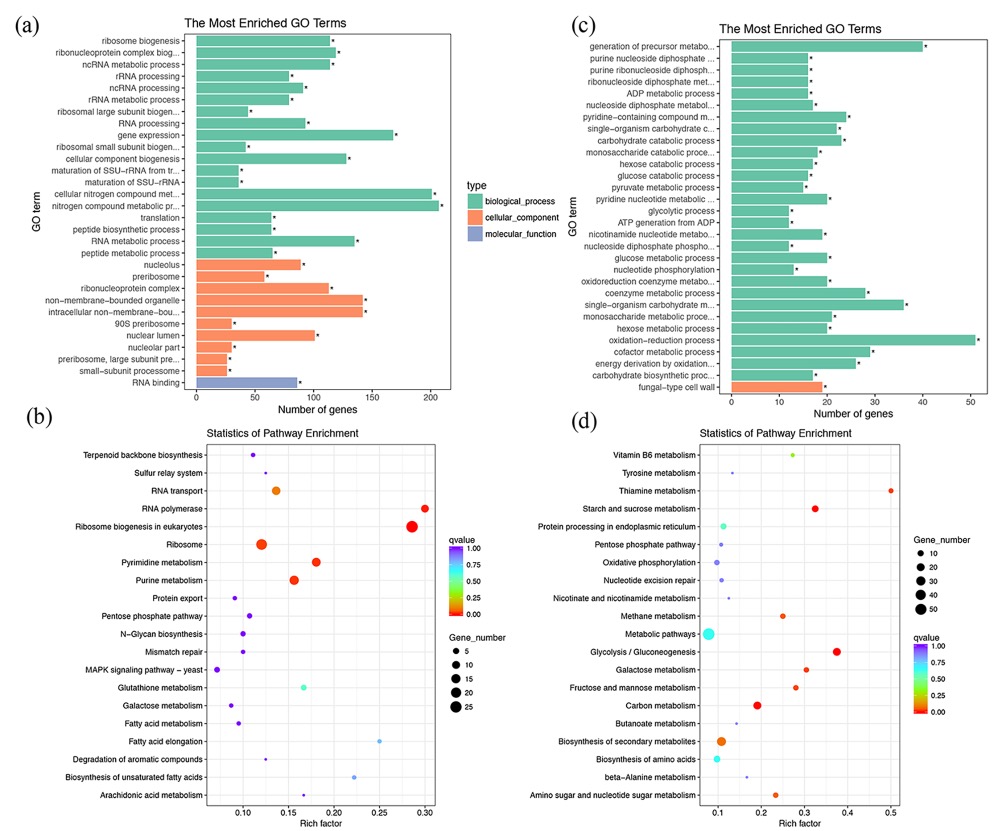
**

**Fig. S4:** Representation of differentially expressed genes in selected GO categories and KEGG pathways in the strain BY4742/I24 after being exposed to multiple inhibitors (0.8 g/L furfural, 3.0 g/L acetic acid and 0.3 g/L phenol) until the middle of the lag phase. (a) The most enriched GO terms of the up-regulated genes. (b) Statistics of pathway enrichment of the up-regulated genes. (c) The most enriched GO terms of the down-regulated genes. (d) Statistics of pathway enrichment of the down-regulated genes.

**Fig. S5**

**
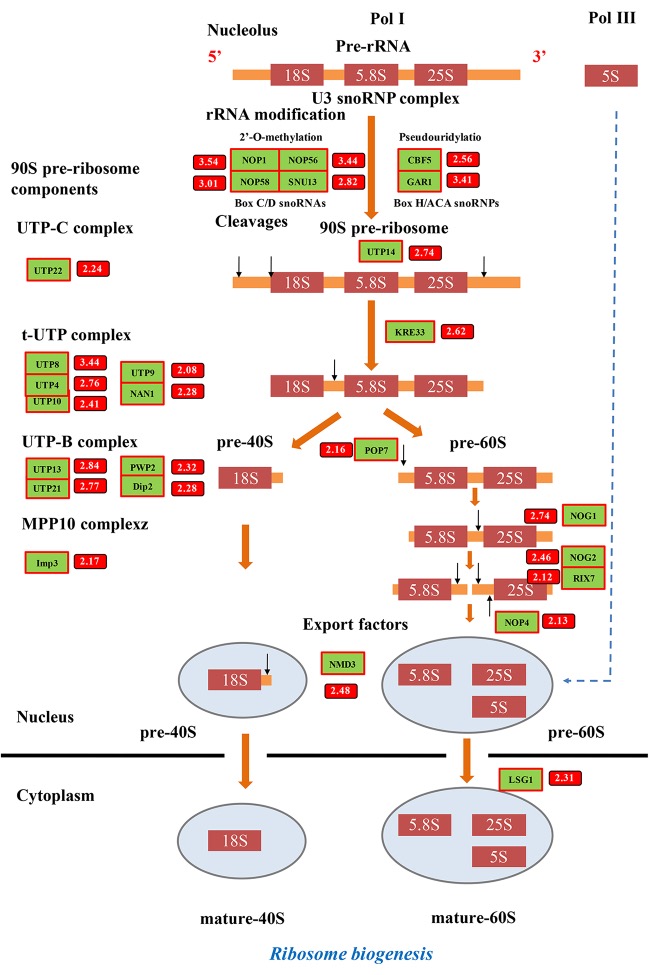
**

**Fig. S5:** Transcriptional profiles of ribosome biogenesis by expressing I24. The strain BY4742/I24 and control strain BY4742/pRS416 were cultured in SC-Ura medium with FAP tolerance. Samples were taken in the middle of the lag phase. Box number exhibits transcriptional changes, which is the foldchange of the transcriptional level of the strain BY4742/I24 to that of the control strain BY4742/pRS416. Up-regulated genes are highlighted in red.

**Fig. S6**


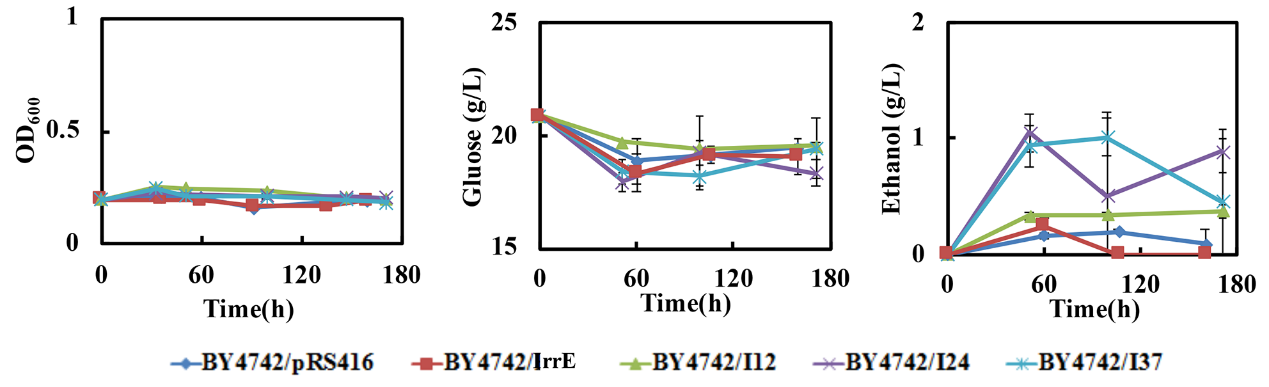


**Fig. S6:** The growth behaviors, glucose consumption, and ethanol production of strains BY4742/pRS416, BY4742/IrrE, BY4742/I12, BY4742/I24, and BY4742/I37 at 38 ℃ in the presence of 0.8 g/L furfural, 3.0 g/L acetic acid, and 0.3 g/L phenol.

**Fig. S7**


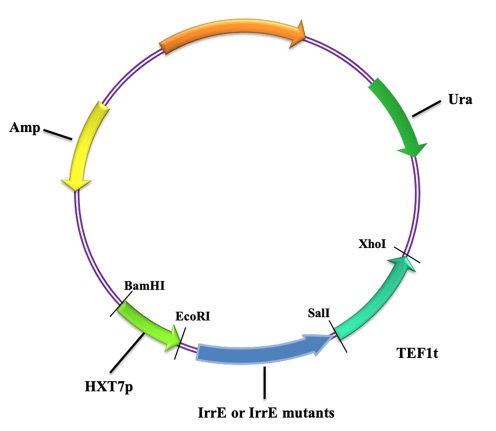


**Fig. S7:** Plasmid map for plasmid pRS416-HXT7p-IrrE-TEF1t and IrrE library construction
